# A new *Hermeuptychia* (Lepidoptera, Nymphalidae, Satyrinae) is sympatric and synchronic with *H. sosybius* in southeast US coastal plains, while another new *Hermeuptychia* species – not *hermes* – inhabits south Texas and northeast Mexico

**DOI:** 10.3897/zookeys.379.6394

**Published:** 2014-02-12

**Authors:** Qian Cong, Nick V. Grishin

**Affiliations:** 1 Howard Hughes Medical Institute; 2 Departments of Biophysics and Biochemistry, University of Texas Southwestern Medical Center, 5323 Harry Hines Blvd, Dallas, TX, USA 75390-9050

**Keywords:** Biodiversity, cryptic species, DNA barcodes, neotropical, satyr, *Hermeuptychia gisella*, *Hermeuptychia cucullina*, *Hermeuptychia sosybius kappeli*, female genitalia

## Abstract

*Hermeuptychia intricata* Grishin, **sp. n.** is described from the Brazos Bend State Park in Texas, United States, where it flies synchronously with *Hermeuptychia sosybius* (Fabricius, 1793). The two species differ strongly in both male and female genitalia and exhibit 3.5% difference in the COI barcode sequence of mitochondrial DNA. Setting such significant genitalic and genotypic differences aside, we were not able to find reliable wing pattern characters to tell a difference between the two species. This superficial similarity may explain why *H. intricata*, only distantly related to *H. sosybius*, has remained unnoticed until now, despite being widely distributed in the coastal plains from South Carolina to Texas, USA (and possibly to Costa Rica). Obscuring the presence of a cryptic species even further, wing patterns are variable in both butterflies and ventral eyespots vary from large to almost absent. To avoid confusion with the new species, **neotype** for *Papilio sosybius* Fabricius, 1793, a common butterfly that occurs across northeast US, is designated from Savannah, Georgia, USA. It secures the universally accepted traditional usage of this name. Furthermore, we find that DNA barcodes of *Hermeuptychia* specimens from the US, even those from extreme south Texas, are at least 4% different from those of *H. hermes* (Fabricius, 1775)—type locality Brazil: Rio de Janeiro—and suggest that the name *H. hermes* should not be used for USA populations, but rather reserved for the South American species. This conclusion is further supported by comparison of male genitalia. However, facies, genitalia and 2.1% different DNA barcodes set *Hermeuptychia* populations in the lower Rio Grande Valley of Texas apart from *H. sosybius*. These southern populations, also found in northeastern Mexico, are described here as *Hermeuptychia hermybius* Grishin, **sp. n.** (type locality Texas: Cameron County). While being phylogenetically closer to *H. sosybius* than to any other *Hermeuptychia* species, *H. hermybius* can usually be recognized by wing patterns, such as the size of eyespots and the shape of brown lines on hindwing. “Intricate Satyr” and “South Texas Satyr” are proposed as the English names for *H. intricata* and *H. hermybius*, respectively.

## Introduction

What could be more exciting than a discovery of a new butterfly species? Perhaps the discovery of a butterfly species in the US that was long overlooked, completely unexpected, and has closest named relatives far away in Bolivia and Brazil. These finds may not be easy to come by, because most of such species are cryptic and appear superficially similar to their more common and well-known relatives. However, DNA-based techniques introduced in taxonomy during the last few decades offer viable tools to facilitate discovery of cryptic species (Bickford et al. 2007).

The genus *Hermeuptychia* was proposed by Forster (1964) on the basis of male genitalia to circumscribe a group of close relatives hardly separable by highly variable wing patterns, but distinct in male genitalia. Lamas (2004) recognized eight named species of *Hermeuptychia* and suggested the existence of several unnamed species in Colombia and Peru. A recent comparative study of DNA barcodes and morphology of male genitalia from all parts of *Hermeuptychia* range revealed congruence between classifications by barcodes and genitalia, hypothesized that *Hermeuptychia gisella* (Hayward, 1957) is a species distinct from *Hermeuptychia cucullina* (Weymer, 1911), and discussed several unnamed species in Brazil (Seraphim et al. 2014). Interestingly, specimens with barcodes and genitalia similar to *Hermeuptychia hermes* (Fabricius, 1775)—type locality Brazil: Rio de Janeiro—were not found north of Costa Rica. Most importantly for this work, Seraphim et al. (2014) outlined several distinct molecular and morphological groups of species, assigned existing names to these groups, illustrated their genitalia and listed genitalia characters in their Table 1. All *Hermeuptychia* specimens from the US (North Carolina, Tennessee and Florida) used by Seraphim et al. (2014) possessed similar DNA barcode sequences and were assigned to morphogroup 4 by male genitalia.

**Table 1.** Data for specimens with DNA sequences used in this study.

Species	Voucher	GenBank	Locality	Date	Collector
*Hermeuptychia sosybius*	NVG-696	KJ025523	OK: Atoka Co., 13 air mi E of Atoka, 34.41186, -95.91044, 225 m	29-Aug-2009	Nick V. Grishin
*Hermeuptychia sosybius*	NVG-1632	KJ025524	TX: Lamar Co., 11.5 air mi NW of Paris, FM1499 @ Sanders Cr., 140 m	25-Apr-1998	Nick V. Grishin
*Hermeuptychia sosybius*	NVG-1630	KJ025525	TX: Marion Co., nr. Carter L., 50 m	28-Sep-1996	Nick V. Grishin
*Hermeuptychia sosybius*	NVG-1633	KJ025526	TX: Marion Co., nr. Carter L., 50 m	29-Sep-1996	Nick V. Grishin
*Hermeuptychia sosybius*	NVG-1606	KJ025527	TX: Wise Co., LBJ National Grassland, 300 m	3-Aug-1998	Nick V. Grishin
*Hermeuptychia sosybius*	NVG-783	KJ025528	TX: Tyler Co., John H. Kirby SF, 40 m	19-Mar-2011	Nick V. Grishin
*Hermeuptychia sosybius*	NVG-784	KJ025529	TX: Tyler Co., John H. Kirby SF, 40 m	19-Mar-2011	Nick V. Grishin
*Hermeuptychia sosybius*	NVG-785	KJ025530	TX: Tyler Co., John H. Kirby SF, 40 m	19-Mar-2011	Nick V. Grishin
*Hermeuptychia sosybius*	NVG-786	KJ025531	TX: Tyler Co., John H. Kirby SF, 40 m	19-Mar-2011	Nick V. Grishin
*Hermeuptychia sosybius*	NVG-1537	KJ025532	TX: Fort Bend Co., Brazos Bend SP, Horseshoe L. tr., 29.38193, -95.61141, 15 m	17-Aug-2013	Nick V. Grishin
*Hermeuptychia sosybius*	NVG-1538	KJ025533	TX: Fort Bend Co., Brazos Bend SP, Horseshoe L. tr., 29.38193, -95.61141, 15 m	17-Aug-2013	Nick V. Grishin
*Hermeuptychia sosybius*	NVG-1539	KJ025534	TX: Fort Bend Co., Brazos Bend SP, Horseshoe L. tr., 29.38193, -95.61141, 15 m	17-Aug-2013	Nick V. Grishin
*Hermeuptychia sosybius*	NVG-1540	KJ025535	TX: Fort Bend Co., Brazos Bend SP, Horseshoe L. tr., 29.38193, -95.61141, 15 m	17-Aug-2013	Nick V. Grishin
*Hermeuptychia sosybius*	NVG-1542	KJ025536	TX: Fort Bend Co., Brazos Bend SP, Horseshoe L. tr., 29.38193, -95.61141, 15 m	17-Aug-2013	Nick V. Grishin
*Hermeuptychia sosybius*	NVG-1543	KJ025537	TX: Fort Bend Co., Brazos Bend SP, Horseshoe L. tr., 29.38193, -95.61141, 15 m	17-Aug-2013	Nick V. Grishin
*Hermeuptychia sosybius*	NVG-1544	KJ025538	TX: Fort Bend Co., Brazos Bend SP, Horseshoe L. tr., 29.38193, -95.61141, 15 m	17-Aug-2013	Nick V. Grishin
*Hermeuptychia sosybius*	NVG-1545	KJ025539	TX: Fort Bend Co., Brazos Bend SP, Horseshoe L. tr., 29.38193, -95.61141, 15 m	17-Aug-2013	Nick V. Grishin
*Hermeuptychia sosybius*	NVG-1546	KJ025540	TX: Fort Bend Co., Brazos Bend SP, Horseshoe L. tr., 29.38193, -95.61141, 15 m	17-Aug-2013	Nick V. Grishin
*Hermeuptychia sosybius*	NVG-1547	KJ025541	TX: Fort Bend Co., Brazos Bend SP, Horseshoe L. tr., 29.38193, -95.61141, 15 m	17-Aug-2013	Nick V. Grishin
*Hermeuptychia sosybius*	NVG-1549	KJ025542	TX: Fort Bend Co., Brazos Bend SP, Horseshoe L. tr., 29.38193, -95.61141, 15 m	17-Aug-2013	Nick V. Grishin
*Hermeuptychia sosybius*	NVG-1550	KJ025543	TX: Fort Bend Co., Brazos Bend SP, Horseshoe L. tr., 29.38193, -95.61141, 15 m	17-Aug-2013	Nick V. Grishin
*Hermeuptychia sosybius*	NVG-1552	KJ025544	TX: Fort Bend Co., Brazos Bend SP, Horseshoe L. tr., 29.38193, -95.61141, 15 m	17-Aug-2013	Nick V. Grishin
*Hermeuptychia sosybius*	NVG-1553	KJ025545	TX: Fort Bend Co., Brazos Bend SP, Horseshoe L. tr., 29.38193, -95.61141, 15 m	17-Aug-2013	Nick V. Grishin
*Hermeuptychia sosybius*	NVG-1557	KJ025546	TX: Fort Bend Co., Brazos Bend SP, nr. Hale L., 29.38008, -95.58473, 16 m	17-Aug-2013	Nick V. Grishin
*Hermeuptychia sosybius*	NVG-1559	KJ025547	TX: Fort Bend Co., Brazos Bend SP, nr. Hale L., 29.38008, -95.58473, 16 m	17-Aug-2013	Nick V. Grishin
*Hermeuptychia sosybius*	NVG-1561	KJ025548	TX: Fort Bend Co., Brazos Bend SP, nr. Hale L., 29.38008, -95.58473, 16 m	17-Aug-2013	Nick V. Grishin
*Hermeuptychia sosybius*	NVG-1562	KJ025549	TX: Fort Bend Co., Brazos Bend SP, nr. Hale L., 29.38008, -95.58473, 16 m	17-Aug-2013	Nick V. Grishin
*Hermeuptychia sosybius*	NVG-1564	KJ025550	TX: Fort Bend Co., Brazos Bend SP, nr. Hale L., 29.38008, -95.58473, 16 m	17-Aug-2013	Nick V. Grishin
*Hermeuptychia sosybius*	NVG-1566	KJ025551	TX: Fort Bend Co., Brazos Bend SP, nr. Hale L., 29.38008, -95.58473, 16 m	17-Aug-2013	Nick V. Grishin
*Hermeuptychia sosybius*	NVG-1567	KJ025552	TX: Fort Bend Co., Brazos Bend SP, nr. Hale L., 29.38008, -95.58473, 16 m	17-Aug-2013	Nick V. Grishin
*Hermeuptychia sosybius*	13385H04	KJ025553	TX: Comal Co., New Braunfels	3-Oct-1981	
*Hermeuptychia sosybius*	13385H11	KJ025554	TX: Williamson Co., Florence	3-Sep-1974	J. Parkinson
*Hermeuptychia sosybius*	13385H03	KJ025555	TX: Uvalde Co., Utopia	10-Jun-1992	D. E. Gaskin & EAL
*Hermeuptychia sosybius*	13385H05	KJ025556	TX: Uvalde Co., Utopia	{9-23}-Sep-1994	D. E. Gaskin & EAL
*Hermeuptychia sosybius*	13385H06	KJ025557	TX: Uvalde Co., Utopia	{9-23}-Sep-1994	D. E. Gaskin & EAL
*Hermeuptychia sosybius*	13385H07	KJ025558	TX: Uvalde Co., Utopia	{13-22}-Apr-1995	D. E. Gaskin
*Hermeuptychia sosybius*	13385H08	KJ025559	TX: Uvalde Co., Utopia	{13-22}-Apr-1995	D. E. Gaskin
*Hermeuptychia sosybius*	13385G12	KJ025560	FL: Highlands Co., Lake Placid, Archbold Biological Station	17-Feb-1985	D. C. Ferguson
Hermeuptychia sosybius*	13386A07	KJ025561	GA: Chatham Co., Savannah	28-Jul-1958	Coll. Gordon B. Small
Hermeuptychia sosybius**	NVG-1845	KJ025562	FL: N of L. Okeechobee	29-Mar-1983	Ralf H. Anken
*Hermeuptychia sosybius*	15609E04	KJ025563	FL: Pinellas Co., St. Petersburg	**3-Nov-1938**	H. E. Wilford
*Hermeuptychia sosybius*	13385G10	KJ025564	SC: Clarendon Co.	**Aug-1909**	
*Hermeuptychia sosybius*	13385H09	KJ025565	TX: Bastrop Co., Bastrop	**prior to 1896**	Collection of O. Meske
*Hermeuptychia sosybius*	13386A01	KJ025566	TX: Guadalupe Co., Seguin	**26-Oct-1905**	F. C. Pratt
*Hermeuptychia sosybius*	13386A04	KJ025567	LA: Jackson Parish, Jonesboro	**4-Jun-1920**	G. W. Rawson
*Hermeuptychia sosybius*	13386A06	KJ025568	LA: Jefferson Parish, Harahan	**11-Aug-1944**	W. D. Field
*Hermeuptychia hermybius*	NVG-1603	KJ025569	TX: Cameron Co., E of Brownsville	17-Mar-2003	Nick V. Grishin
*Hermeuptychia hermybius*	NVG-1607	KJ025570	TX: Cameron Co., E of Brownsville	18-Jan-2003	Nick V. Grishin
*Hermeuptychia hermybius*	NVG-1609	KJ025571	TX: Cameron Co., E of Brownsville	30-Mar-2003	Nick V. Grishin
*Hermeuptychia hermybius*	NVG-1610	KJ025572	TX: Cameron Co., E of Brownsville	9-Mar-2003	Nick V. Grishin
*Hermeuptychia hermybius*	NVG-1611	KJ025573	TX: Cameron Co., E of Brownsville	14-Mar-2003	Nick V. Grishin
*Hermeuptychia hermybius*	NVG-1612	KJ025574	TX: Cameron Co., E of Brownsville	16-Mar-2003	Nick V. Grishin
*Hermeuptychia hermybius*	NVG-1628	KJ025575	TX: Cameron Co., E of Brownsville	19-Oct-1997	Nick V. Grishin
*Hermeuptychia hermybius*	NVG-1695	KJ025576	TX: Hidalgo Co., 1.5 air mi SE of Relampago, Rio Rico Rd., 26.07, -97.891, 21 m	19-Oct-2013	William R. Dempwolf
*Hermeuptychia hermybius*	NVG-1698	KJ025577	TX: Hidalgo Co., 1.5 air mi SE of Relampago, Rio Rico Rd., 26.07, -97.891, 21 m	19-Oct-2013	William R. Dempwolf
*Hermeuptychia hermybius*	NVG-1699	KJ025578	TX: Hidalgo Co., 1.5 air mi SE of Relampago, Rio Rico Rd., 26.07, -97.891, 21 m	19-Oct-2013	William R. Dempwolf
*Hermeuptychia hermybius*	NVG-1712	KJ025579	TX: Starr Co., Rio Grande City, Fort Ringgold, 26.3707, -98.8064, 45 m	20-Oct-2013	William R. Dempwolf
*Hermeuptychia hermybius*	NVG-1714	KJ025580	TX: Starr Co., Rio Grande City, Fort Ringgold, 26.3707, -98.8064, 45 m	20-Oct-2013	William R. Dempwolf
*Hermeuptychia hermybius*	NVG-1726	KJ025581	TX: Starr Co., Roma, S of Roma International Bridge, 26.4035, -99.0175, 50 m	20-Oct-2013	William R. Dempwolf
*Hermeuptychia hermybius*	NVG-1727	KJ025582	TX: Starr Co., Roma, S of Roma International Bridge, 26.4035, -99.0175, 50 m	20-Oct-2013	William R. Dempwolf
*Hermeuptychia hermybius*	NVG-1735	KJ025583	TX: Starr Co., 0.5 mi S of Fronton, 26.399, -99.085, 50 m	20-Oct-2013	William R. Dempwolf
*Hermeuptychia hermybius*	NVG-1737	KJ025584	TX: Starr Co., 0.5 mi S of Fronton, 26.399, -99.085, 50 m	20-Oct-2013	William R. Dempwolf
*Hermeuptychia hermybius*	NVG-1747	KJ025585	TX: Starr Co., Salineno @ Rio Grande, 26.51463, -99.11633, 53 m	23-Oct-2013	William R. Dempwolf
*Hermeuptychia hermybius*	NVG-1635	KJ025586	TX: Zapata Co., San Ygnacio @ Rio Grande, 92 m	7-Oct-2007	Nick V. Grishin
*Hermeuptychia hermybius*	13385H10	KJ025587	TX: Webb Co., Laredo	15-Apr-1949	E. L. Todd
*Hermeuptychia intricata*	NVG-1541	KJ025588	TX: Fort Bend Co., Brazos Bend SP, Horseshoe L. tr., 29.38193, -95.61141, 15 m	17-Aug-2013	Nick V. Grishin
*Hermeuptychia intricata*	NVG-1548	KJ025589	TX: Fort Bend Co., Brazos Bend SP, Horseshoe L. tr., 29.38193, -95.61141, 15 m	17-Aug-2013	Nick V. Grishin
*Hermeuptychia intricata*	NVG-1551	KJ025590	TX: Fort Bend Co., Brazos Bend SP, Horseshoe L. tr., 29.38193, -95.61141, 15 m	17-Aug-2013	Nick V. Grishin
*Hermeuptychia intricata*	NVG-1554	KJ025591	TX: Fort Bend Co., Brazos Bend SP, nr. Hale L., 29.38008, -95.58473, 16 m	17-Aug-2013	Nick V. Grishin
*Hermeuptychia intricata*	NVG-1555	KJ025592	TX: Fort Bend Co., Brazos Bend SP, nr. Hale L., 29.38008, -95.58473, 16 m	17-Aug-2013	Nick V. Grishin
*Hermeuptychia intricata*	NVG-1556	KJ025593	TX: Fort Bend Co., Brazos Bend SP, nr. Hale L., 29.38008, -95.58473, 16 m	17-Aug-2013	Nick V. Grishin
*Hermeuptychia intricata*	NVG-1558	KJ025594	TX: Fort Bend Co., Brazos Bend SP, nr. Hale L., 29.38008, -95.58473, 16 m	17-Aug-2013	Nick V. Grishin
Hermeuptychia intricata*	NVG-1560	KJ025595	TX: Fort Bend Co., Brazos Bend SP, nr. Hale L., 29.38008, -95.58473, 16 m	17-Aug-2013	Nick V. Grishin
*Hermeuptychia intricata*	NVG-1563	KJ025596	TX: Fort Bend Co., Brazos Bend SP, nr. Hale L., 29.38008, -95.58473, 16 m	17-Aug-2013	Nick V. Grishin
*Hermeuptychia intricata*	NVG-1565	KJ025597	TX: Fort Bend Co., Brazos Bend SP, nr. Hale L., 29.38008, -95.58473, 16 m	17-Aug-2013	Nick V. Grishin
*Hermeuptychia intricata*	NVG-1629	KJ025598	TX: San Jacinto Co., Sam Houston NF, USF217 @ Big Creek, 58 m	12-Apr-1998	Nick V. Grishin
*Hermeuptychia intricata*	NVG-1631	KJ025599	TX: Brazoria Co., Bar-X Ranch, Rd. 971N, 29.13252, -95.58340, 7 m	4-Mar-2000	Nick V. Grishin
*Hermeuptychia intricata*	13385G07	KJ025600	SC: Charleston Co., McClellanville, Wedge Plantation	6-Apr-1970	D. C. Ferguson
*Hermeuptychia intricata*	13385H01	KJ025601	FL: Alachua Co., Gainesville	12-Mar-1983	Scott W. Gross
*Hermeuptychia intricata*	13385H02	KJ025602	FL: “Putnam Co | Shell Bluff Landing”	29-Sep-1985	George Balogh
*Hermeuptychia intricata*	13386A03	KJ025603	LA: Jefferson Parish, Harahan	28-Jun-1944	W. D. Field
*Hermeuptychia intricata*	13385G08	KJ025604	SC: Clarendon Co.	**9-Aug-1898**	
*Hermeuptychia intricata*	13385G09	KJ025605	SC: Clarendon Co.	**Aug-1910**	
*Hermeuptychia intricata*	13385G11	KJ025606	SC: Clarendon Co.	**Aug-1910**	
*Hermeuptychia intricata*	13386A02	KJ025607	“Flatbush LI”	**prior to 1941**	G. P. Engelhardt Coll.
*Hermeuptychia sosybius*	DNA-ATBI-0799	GU089906*	NC: Swain Co., AN9, Smokemont Stables, 35.5504, -83.3084	20-Jul-2004	R. M. Pyle
*Hermeuptychia sosybius*	NSHer-EUA07	KF466083*	TN: Rutheford Co., 35.70, -86.33	2009	A. V. Z. Brower
*Hermeuptychia sosybius*	NSHer-EUA08	KF466084*	TN: Rutheford Co., 35.70, -86.33	2009	A. V. Z. Brower
*Hermeuptychia sosybius*	DNA-ATBI-0847	GU089907*	TN: Blount Co. AN2, Cades Cove, along Forge Cr. Rd. 35.583, -83.838	20-Jul-2004	R. M. Pyle
*Hermeuptychia sosybius*	DNA-ATBI-0848	GU089908*	TN: Blount Co. AN2, Cades Cove, along Forge Cr. Rd. 35.583, -83.838	20-Jul-2004	R. M. Pyle
*Hermeuptychia sosybius*	DNA-ATBI-0849	GU089909*	TN: Blount Co. AN2, Cades Cove, along Forge Cr. Rd. 35.583, -83.838	20-Jul-2004	R. M. Pyle
*Hermeuptychia sosybius*	DNA-ATBI-4110	GU088393*	TN: Sevier Co., Lyon Spring Rd., 35.6, -83.4	22-May-2005	Segebarth
*Hermeuptychia sosybius*	DNA-ATBI-4109	GU088394*	TN: Sevier Co., Lyon Spring Rd., 35.6, -83.4	22-May-2005	Segebarth
*Hermeuptychia sosybius*	NSHer-EUA02	KF466080*	FL: Gainesville, 29.65, -82.32	Apr-2009	K. R. Willmott
*Hermeuptychia sosybius*	NSHer-EUA03	KF466081*	FL: Gainesville, 29.65, -82.32	Apr-2009	K. R. Willmott
*Hermeuptychia sosybius*	NSHer-EUA06	KF466082*	FL: Gainesville, 29.65, -82.32	Apr-2009	K. R. Willmott
*Hermeuptychia cucullina*	NSHer-PE03	KF466142*	Peru		C Peña
*Hermeuptychia gisella*	NSHer-J29	KF466092*	Brazil: São Paulo, Serra do Japí, Jundiaí, -23.22, -46.92	26-Feb-2008	P. E. C. Peixoto
*Hermeuptychia atalanta*	R10_CA_SP	JN109040*	Brazil: São Paulo, Ribeirão Cachoeira, Campinas		
*Hermeuptychia hermes*	NSHer-MG08	KF466108*	Brazil: Minas Gerais, Serra do Cipó, Jaboticatubas, -18.20, -43.50	Dec-2005	A. R. M. Silva
*Hermeuptychia maimoune*	NSHer-CO04	KF466021*	Colombia, Meta, Bosque Bavaria, 4.18, -73.65	8-Oct-2006	M. A. Marín
*Hermeuptychia pimpla*	CP04-10	GU205843*	Peru: Quebrada Siete Jeringas		
*Hermeuptychia harmonia*	CP06-93	GU205842*	Peru: Quebrada Siete Jeringas		
*Hermeuptychia fallax*	NSHer-J17	KF466089*	Brazil: São Paulo, Serra do Japí, Jundiaí, -23.22, -46.92	26-Feb-2008	P. E. C. Peixoto
*Megisto cymela*	DNA-ATBI-4114	GU088434*	TN: Sevier Co., Lyon Spring Rd., 35.6, -83.4	22-May-2005	Segebarth
*Hermeuptychia intricata*?	DNA96-016	AY508548*	Costa Rica: Puntarenas Province		

Abbreviations: SP State Park; L. Lake; Cr Creek tr. trail; nr. near; Co. County; NF National Forest; SF State Forest Rd. Road * after the species name indicates primary type specimen, ** is *Hermeuptychia hermes kappeli* holotype * after the GenBank number indicates that it was retrieved from GenBank, all other sequences were determined by us in this study Only DNA ID tags were obtained for the oldest specimens and their dates are shown in **bold font**.

Here, we show that two distinct species from two different morphogroups as defined by Seraphim et al. (2014) fly together at the same location in Texas on the same day. These two species possess very different genitalia in both sexes and 3.5% difference in DNA barcodes. One of these species has traditionally been called *Hermeuptychia sosybius* (Fabricius, 1793) and the neotype for it is designated herein. The second species is apparently new, and is from the same molecular group with South American species *Hermeuptychia cucullina* (from Peru and Bolivia) and *Hermeuptychia gisella* (from Bolivia and Brazil). This new species is described, discussed and illustrated. Furthermore, we find that DNA barcodes of *Hermeuptychia* from the lower Rio Grande Valley region of Texas (Webb, Zapata, Starr, Hidalgo, and Cameron Counties) form a tight cluster and differ by at least 2% from the barcodes of over 50 *Hermeuptychia sosybius* specimens (divergence average 0.09%, standard deviation 0.19%, maximum below 1%) across its range from North Carolina to Texas (south to Uvalde, Comal, Guadalupe and Brazoria Counties). In addition to DNA barcodes, these south Texas *Hermeuptychia* populations differ from *Hermeuptychia sosybius* by wing patterns and male genitalia (subtly, but quantifiably) and are described here as another new species, bringing the total count of USA *Hermeuptychia* species to three.

## Materials and methods

Specimens used in this study were collected in the field under the permit #08-02Rev from Texas Parks and Wildlife Department to NVG, and inspected in the following collections: Texas A&M University Insect Collection, College Station, TX (TAMU); National Museum of Natural History, Smithsonian Institution, Washington, DC (USNM); Natural History Museum, London, UK (BMNH). Standard entomological techniques were used for dissection (Robbins 1991), i.e. abdomen was broken off, soaked for 40 minutes (or until ready) in 10% KOH at 60 °C (or overnight at room temperature), dissected, and subsequently stored in a small glycerol-filled vial on the pin under the specimen. Genitalia and wing venation terminology follows Steinhauser (1981). Length measurements are in metric units and were made from photographs of specimens taken with a scale and magnified on a computer screen. Photographs of immature stages were taken by NVG using Minolta Maxxum 500si 35mm SLR film camera through a 90 mm f/2.8 Tamron SP AF Macro lens (for smaller objects additionally with a Phoenix C/D7 AF 2X Teleconverter) on Kodachrome 25 or Fuji Velvia 50 slide films and slides were scanned using Nikon Super CoolScan 5000 ED film scanner. Photographs of specimens were taken with a Nikon D800 camera through a 105 mm f/2.8G AF-S VR Micro-Nikkor lens; dissected genitalia were photographed in glycerol with a Nikon D200 camera without a lens and through microscopes at 4×–5× magnification. Images were assembled and edited in Photoshop CS5.1. Genitalic photographs were taken in several focus slices and stacked in Photoshop to increase depth of field.

Two legs (cut with scissors into tiny pieces in lysis buffer) of freshly collected specimens, or two legs that were removed from freshly collected specimens and preserved in alcohol for several years, or an abdomen (dropped into lysis buffer as a whole, and after overnight incubation at 56 °C transferred into 10% KOH for genitalia dissection) of older specimens were used to extract genomic DNA with QIAGEN DNeasy blood and tissue kit complemented with EconoSpin columns from Epoch, or Macherey-Nagel (MN) NucleoSpin® tissue kit following the manufacturer’s protocol. Genomic DNA was eluted in a total volume of 120-150 μl QIAGEN AE buffer (concentration of DNA as measured by Promega QuantiFluor® dsDNA System was from 0.01 to 2.5 ng/μl for legs and from 0.005 to 30 ng/μl for abdomens, depending on specimen age and storage conditions) and was stored at -20 °C.

PCR was performed using Invitrogen AmpliTaq Gold 360 master mix in a 20 μl total volume containing less than 10 ng of template DNA and 0.5 μM of each primer. For legs from freshly collected specimens or those preserved in alcohol, the following primers were used to obtain the complete barcode: LepF: 5’-TGTAAAACGACGGCCAGTATTCAACCAATCATAAAGATATTGG-3'and LepR: 5’-CAGGAAACAGCTATGACCTAAACTTCTGGATGTCCAAAAAATCA-3’. For older specimens the following pairs of primers were used: sCOIF (forward, 5’-ATTCAACCAATCATAAAGATATTGG-3’) – smCOIR (reverse, 5’-CCTGTTCCAGCTCCATTTTC-3’) and bat-smCOIF (forward, 5’-GCTTTTCCTCGTATAAATAATA-3’) – sCOIR (reverse, 5’-TAAACTTCTGGATGTCCAAAAAATCA-3’), to amplify barcode in two overlapping segments (307, 408 bp).

The barcodes of the *Hermeuptychia sosybius* neotype (designated below) and *Hermeuptychia hermes kappeli* Anken, 1993 holotype were amplified in four overlapping segments with the following four pairs of *Hermeuptychia*-specific primers: styr-COIF (forward, 5’-CAACCAATCATAAAGATATTGGAAC-3’) – styr-bCOIR (reverse, 5’-AAAATTATAATAAAAGCATGRGCTGT-3’), styr-bCOIF (forward, 5’-YCCAGGATTTTTAATTGGAGATG-3’) – styr-mCOIR (reverse, 5’-CCTGTYCCACTTCCATTTTCTAC-3’), styr-mCOIF (forward, 5’-TTTTGATTATTACCYCCATCTTT-3’) – styr-eCOIR (reverse, 5’-TTCCTACAGCTCAAATAAATAAAGG-3’), and styr-eCOIF (forward, 5’-TTCATTTAGCTGGAATTTCWTCAA-3’) – sCOIR (reverse, 5’-TAAACTTCTGGATGTCCAAAAAATCA-3’).

For very old specimens (e.g., from 1898 to 1944), amplification of longer DNA segments failed. To obtain their sequences for identification, we developed *Hermeuptychia*-specific primers for very short, about 100 bp fragments, which we call ID tags. Two regions, in which the three USA *Hermeuptychia* species differ from each other the most, were selected and the following primers were designed: styr-ID1F (forward, 5’-TTGAGCAGGAATAATTGGWACAT-3’) – styr-ID1R (reverse, 5’-AAAAGCATGRGCTGTAACAA-3’) and styr-ID2F (forward, 5’-TTGGAGGATTTGGTAATTGACTT-3’) – styr-ID2R (reverse, 5’-AAAGATGGRGGTAATAATCAAAAT-3’) to amplify 75 and 56 bp sequence from the specimen (together with both primers, the actual products are 118 and 103 bp).

These primers yielded clear DNA sequence traces (Fig. 65) for 11 out of 12 specimens. The failed traces from DNA voucher 13386A05 showed signs of contamination (i.e., multiple peaks at many positions, probably not even a *Hermeuptychia* sequence) and were inconclusive. Genitalia, however, offered unambiguous identification of this specimen. We did not pursue re-extraction of DNA from the 13386A05 specimen and were satisfied with higher than 90% success rate (11 out of 12) of this method. The oldest specimens from 1898 and likely prior to 1896 (date not specified on the label of the second specimen, and 1896 is the date of collection donation) yielded excellent traces (e.g., Fig. 65). 6, 1 and 4 specimens of each of the three species were sequenced. For DNA extraction and PCR reactions, they were intermixed and ordered not by species, but as they were placed in USNM collection by curators who did not suspect the presence of more than one species (i.e. semi-randomly, according to DNA voucher numbers assigned to them). Because cross-contamination frequently happens between adjacent specimens, this arrangement alleviates biasing DNA conclusions on the basis of our genitalia and wing pattern-based identification. I.e., if adjacent specimens are the same species (and thus are likely to possess the same DNA barcode), it is more difficult to detect cross-contamination from neighbors. However, if they are different species, disagreement between genitalia-based identification and DNA-based identification would raise suspicions of cross-contamination. All 11 successful DNA identifications were invariably the same as identifications on the basis of genitalia and wing patterns (the voucher 15609E04, Fig. 44, lacked abdomen), and agreed with geographic distribution of these species.

PCR reaction was cleaned up by enzymatic digestion for the whole barcode amplifications of DNA from freshly collected or alcohol preserved specimens and ID tag amplification of old specimens with 4 μl Shrimp Alkaline Phosphatase (20 U/μl) and 1 ul Exonuclease I (1 U/μl) from New England Biolabs. For older specimens that are barcoded in multiple segments, due to the frequent presence of primer dimers and other short non-specific PCR products, Agencourt Ampure XP beads or Invitrogen E-Gel® EX Agarose Gels (followed by Zymo gel DNA recovery kit) were used to select the DNA products of expected length. Sequences were obtained using the M13 primers (for amplification from LepF and LepR primers): 5’-TGTAAAACGACGGCCAGT-3'or 5’-CAGGAAACAGCTATGACC-3'or with primers used in PCR. For the ID tags, PCR products were sequenced in both directions. Sanger sequencing was performed with Applied Biosystems Big Dye Terminator 3.1 kit on ABI capillary instrument in the DNA Sequencing Core Facility of the McDermott Center at UT Southwestern. The resulting sequence traces were proofread in FinchTV <http://www.geospiza.com/Products/finchtv.shtml>. We obtained complete or partial DNA barcode sequences from 85 *Hermeuptychia* specimens. Sequences and accompanying specimen data were submitted to GenBank and received accession numbers KJ025523–KJ025607. Data about these specimens are provided in Table 1.

Additional DNA sequences were downloaded from GenBank <http://genbank.gov/> using accession numbers provided in Seraphim et al. (2014) or were found by BLAST <http://blast.ncbi.nlm.nih.gov/> searches using sequences obtained by us to query “nr/nt” database. Information about specimens with sequences used in this study is in Table 1. All sequences were aligned manually since they matched throughout their length without insertions or deletions, and analyzed using the Phylogeny.fr server at <http://www.phylogeny.fr/> with default parameters (Dereeper et al. 2008), namely, Kimura 2-parameters model (Kimura 1980) was used to compute evolutionary distances from aligned DNA sequences and BioNJ (Gascuel 1997) algorithm was used to build trees.

## Results and discussion

Taxonomic status of various *Hermeuptychia* populations in Texas has been puzzling (Miller and Brown 1981, Pelham 2008). Some authors treated them as conspecific with eastern USA populations, either under the name *Hermeuptychia sosybius* (Opler and Malikul 1992, Allen 1997, Glassberg et al. 2000, Opler and Warren 2002, Glassberg 2007) or *Hermeuptychia hermes* (Howe 1975, Opler and Krizek 1984, Scott 1986, Neck 1996). Others apparently assigned more southern populations to *Hermeuptychia hermes*, reserving the name *Hermeuptychia sosybius* for eastern butterflies (Miller and Brown 1981, brief comment in Neck 1996, Pelham 2008, Warren et al. 2013).

As a part of a barcoding exercise to shed some light on taxonomy of *Hermeuptychia*, we obtained DNA sequences from several samples across Texas. The results were not as expected. In fact, populations from extreme south Texas with the small eyespots phenotype characteristic of *Hermeuptychia hermes* revealed barcodes more similar to those across eastern US. Genitalic examinations showed that even specimens from Tamaulipas and San Luis Potosí, Mexico possessed characters of morphogroup 4 (i.e. the one that includes *Hermeuptychia sosybius*) from Seraphim et al. (2014).

However, much to our surprise, several specimens from southeast (but not southernmost) Texas, namely from the Brazos Bend State Park in Fort Bend County near Houston, possessed barcodes 3.5% different from those of all other USA populations and, as found by BLAST (Altschul et al. 1990), more than 2% different from all other available sequences (except one, discussed below) in GenBank (Benson et al. 2013). Both males and females were in the sample with the unusual barcodes.

Suspecting DNA introgression, similar to that reported by Zakharov et al. (2009), or some yet unexplained irregularities with barcodes, we critically inspected genitalia of these butterflies. Even more surprisingly, both male and female genitalia of the specimens with unusual barcodes differed profoundly from those with classic morphogroup 4 (suggested *Hermeuptychia sosybius*) barcodes, and male genitalia were more similar to morphogroups 5, 6 and possibly 7 of Seraphim et al. (2014), differing in certain details from all of them. The morphogroups 5 and 6 included specimens from Peru and south Brazil and were associated with the names *Hermeuptychia cucullina* (Weymer, 1911) (type locality: Bolivia) and *Hermeuptychia gisella* (Hayward, 1957), *reinstated status* (type locality: Bolivia) per data provided by Seraphim et al. (2014). Morphogroup 7 referred to an unnamed phenotype from South Brazil.

Apparently, in Fort Bend County, Texas there exist two sympatric and synchronic *Hermeuptychia* species (collected on the same day at exactly the same spot!), one from morphogroup 4 and the other one more similar to morphogroups 5, 6 & 7. Interestingly, a possible closest named relative of this second species is either *Hermeuptychia gisella* or *Hermeuptychia cucullina*, documented from Bolivia and central to southeastern Brazil. The situation might be analogous to another butterfly recently described from the US, *Strymon solitario* Grishin & Durden, 2012, whose possible sibling is *Strymon jacqueline* Nicolay & Robbins, 2005 from Peru (Grishin and Durden 2012).

### Historical investigations into *Papilio sosybius* Fabricius, 1793

**Figures 1–9.**
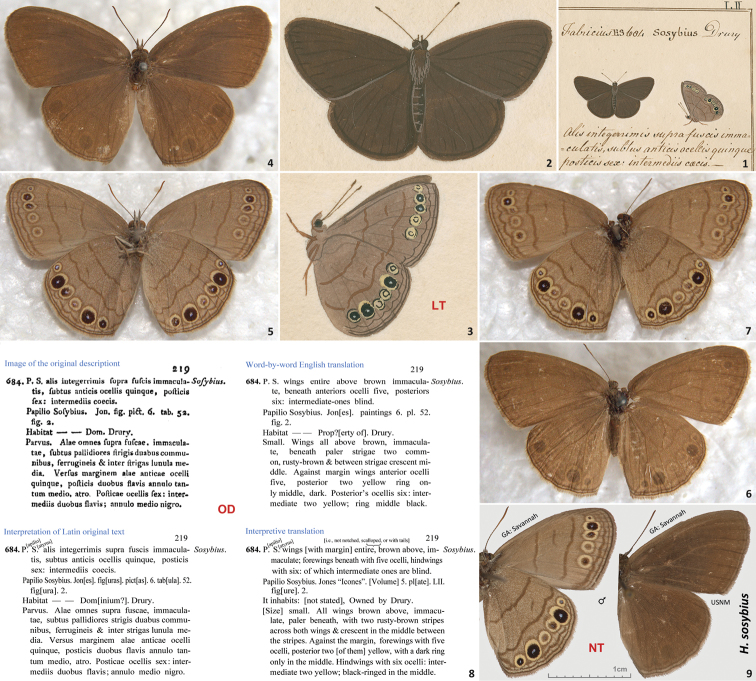
Historical illustrations and specimens of *Hermeuptychia sosybius*, its original description, and neotype. **1–3** Illustration of *Hermeuptychia sosybius* syntype(s) by William Jones [1745–1818] from an unpublished book called the “Icones” (Vane-Wright 2010), currently in Oxford University Museum of Natural History, UK (Smith 1986). **1** shows the upper right quadrant of the plate LII from Volume 5 **2, 3** are magnified cropped images off this plate showing dorsal and ventral aspects, respectively; ventral image (**3**) is rotated clockwise for the ease of comparison with specimens. The specimen with ventral side illustrated (**3** and on the right in **1**) is designated as the lectotype herein and is apparently lost **4**–**7** Two possible syntypes of *Hermeuptychia sosybius* from the Macleay collection (Macleay Museum, The University of Sydney, Australia). Neither specimen bears any labels **4, 6** show dorsal aspect and **5, 7** show ventral aspect **8** Original description of *Hermeuptychia sosybius* and its translations. Note that the Jones illustrations of *Hermeuptychia sosybius* are currently bound within Volume 5, and not 6 as per description **9** Neotype of *Hermeuptychia sosybius* (designated herein, also see Figs 10–11, genitalia Fig. 62p, DNA barcode tree Fig. 66b), in USNM collection, from USA: Georgia: Chatham Co., Savannah, 28-Jul-1958, leg. G. B. Small, genitalia NVG131102-61, DNA voucher 13386A07, GenBank accession for mitochondrial DNA COI barcode KJ025561. Scale bar refers to **9** only, other images are scaled approximately. Images **1**–**3** are copyright of Oxford University Museum of Natural History, UK (used with permission), and images **4**–**7** are copyright of Macleay Museum, The University of Sydney, Australia and are photographed by Robert Blackburn (used with permission).

The two *Hermeuptychia* species from east Texas are markedly different in genitalia of both sexes and in DNA barcodes. However, upon close inspection of wing patterns, we failed to find strong diagnostic differences that would hold against individual variation. Searching for additional specimens revealed the presence of both species across the eastern US from Texas to Florida and South Carolina, but didn’t reveal obvious wing pattern differences either. This posed a problem with the taxonomic identity of these two species, as it was uncertain which one, if any, is *Hermeuptychia sosybius* described by Fabricius (1793: 219). In his brief description, Fabricius referenced unpublished drawings (“Icones”) by William Jones (Vane-Wright 2010): “Jon. fig. pict. 6. tab. 52. fig. 2.” and Drury specimens, but did not state the locality these specimens came from (Figs 1–3, 8). The specimens used by Jones to sketch from and those in the Drury collection (the same specimens?) are *Hermeuptychia sosybius* syntypes. With the help of Kathleen Santry (Head of Archival Collections), we obtained high resolution digital images of the *Hermeuptychia sosybius* drawing by Jones from the Hope Library, Oxford University Museum of Natural History (Oxford, UK). The images show a dorsal side of a specimen on the left, which is uniformly dark-brown; and a ventral side of a specimen on the right (Figs 1–3). Consistent with the Fabricius description (Fig. 8), the ventral surface of wings is paler-brown, with darker brown submedial, postmedial, sinuous submarginal and marginal lines across both wings and end-of-cell dark-brown dash on each wing. Submarginal eyespots are large (compared to *Hermeuptychia hermes*): 5 on the forewing, the 2^nd^ and 3^rd^ from the costa are larger, black-ringed and pupiled; 6 on the hindwing, the 2^nd^, 5^th^ and 6^th^ from the costa are larger, black-ringed and pupilled, 2^nd^ and 5^th^ being the largest. Generally, this wing pattern is consistent with both *Hermeuptychia* species from southeast Texas.

We have taken the following steps to trace the type specimens of *Hermeuptychia sosybius*. First, we studied relevant publications. For instance, Zimsen (1964: 561) specifies for “Papilio Sosybius”: “»... Dom. Drury« – “ with no specimen location mention after the dash. In contrast, for “Papilio Hermes”, Zimsen (1964: 514) lists “»in Brasilia Mus. Banks«,... – London 1 specimen.” Indeed, there is presently a specimen presumed to be *Hermeuptychia hermes* type in Banks collection in BMNH (see images in Warren et al. 2013). Miller and Brown (1981: 191) state “Type lost, a Drury specimen.” Pelham (2008: 404) echoes: “Type(s) probably lost.”

Second, we consulted knowledgeable historians and scholars of Lepidoptera. John V. Calhoun kindly provided the following information: Drury’s collection was sold at auctions and the catalogs of sales did not list specimens of *Hermeuptychia sosybius*. However, species names for many sold specimens were not given. It is possible that the types of *Hermeuptychia sosybius* were acquired by Macleay and are in the Macleay Museum (Sydney, Australia). However, even if *Hermeuptychia sosybius* specimens could be found in the Macleay collection, it will be nearly impossible to figure out which (if any) served as types. Gerardo Lamas (pers. comm.) was not able to trace *Hermeuptychia sosybius* syntypes in his comprehensive search for the primary type specimens of all Neotropical butterflies, and expressed an opinion that it would be very difficult to support the status of any found specimens as syntypes.

Nevertheless, as a third step, we contacted the Macleay Museum staff with a request to search for specimens similar to those illustrated by Jones in the Macleay collection. After extensive search of the Macleay holdings (housed in two places), Robert Blackburn, armed with the Jones illustrations and photographs of *Hermeuptychia sosybius* specimens, was able to find four *Hermeuptychia* specimens of potential interest. According to Mr. Blackburn (pers. comm.), “the history of these 4 is hard [to determine] due to the absolute lack of labels. Much of the material in these drawers came from a mixture of sources, between William Sharp Macleay’s trading network of entomologists and Alexander Macleay’s purchases at auctions. I think that butterflies like these would be most likely to be Alexander Macleay purchases, and probably came through the purchase of Dru Drury’s collections at auction. I think it’s absolutely possible that they are 1780’s specimens, maybe even through John Abbot, as many of the other butterflies in these drawers are labelled ‘Georgia’.” Two of these (Figs 4–7) would be identifiable as *Hermeuptychia sosybius* by facies. Unfortunately, neither specimen bears any labels and it will be very difficult to find supporting evidence that these are indeed syntypes. Even if these specimens are from the Drury collection, since Drury exchanged material, it is impossible to know that these are the original specimens, or the ones acquired after the *Hermeuptychia sosybius* description.

Next, we compared these specimens with the Jones illustrations. The wing pattern and shape of the specimen with abdomen intact (female, Figs 6–7) do not agree closely with the Jones illustrations (Figs 1–3). Most notably, Jones’s illustration of the ventral aspect (Fig. 3) shows two forewing eyespots with strongly developed black rings (near the apex, 2^nd^ and 3^rd^ from the costa), and the specimen has only one (2^nd^ from the costa, the 3^rd^ eyespot entirely lacks black and is more similar to the two posterior eyespots, Fig. 7). The postmedial dark line on ventral hindwing is shaped differently. e.g., it is directed basad near costa in the illustration and is directed distad in the specimen. Other differences in details of placement and shape of eyespots and dark lines are equally obvious, and it is not likely that this specimen was the model for the Jones illustration.

The specimen lacking the abdomen (Figs 4–5) is more similar to the specimen(s) illustrated by Jones, i.e. both 2^nd^ and 3^rd^ eyespots on the forewing are black-ringed and the postmedial hindwing line (slightly) bends basad at costa. However, it seems to be mounted differently than the Jones’s dorsal image shows, i.e. the hindwings that are lowered on the Jones image and touch each other with inner margins, are widely apart in the specimen (Fig. 4). Ventral patterns (in case Jones image Fig. 3 depicts a different specimen from that shown on dorsal image Fig. 2) also differ in detail. In particular, the 3^rd^ hindwing eyespot from the costa lacks black and is more similar to the 4^th^ from costa eyespot in the illustration, but is clearly black-ringed and larger than the 4^th^ eyespot in the specimen (Fig. 5). The submedial and postmedial dark lines on both wings are farther apart in the illustration than in the specimen. The postmedial dark line is strongly bent, directed basad and reaches the hindwing inner margin at an angle in the illustration (more similar to the specimen illustrated in Fig. 47), but is almost perpendicular to the inner margin near the tornus in the specimen. In our opinion, it is not very likely that these obvious pattern differences are caused by inaccuracy of the Jones illustration, in part because we see *Hermeuptychia* specimens (e.g. Fig. 47) that are more similar in such patterns to the Jones illustration than the specimen in Fig. 5. We see that *Hermeuptychia* specimens with the characters illustrated by Jones exist, and it seems more likely that their characters were illustrated, rather that invented by Jones. Therefore, we conclude that neither of the specimens from the Macleay collection is the one illustrated by Jones. John V. Calhoun who has vast experience dealing with the analysis of historical illustrations agrees with this opinion (pers. comm.).

To stabilize nomenclature, similarly to Calhoun (2006), we designate the specimen with ventral aspect illustrated by Jones in Volume 5, plate LII (second species illustrated on this plate), topmost image on the right (reproduced here as Fig. 3) in his unpublished manuscript known as “Icones” (Vane-Wright 2010) and referred to as “Jon. fig. pict. 6. tab. 52. fig. 2.” by Fabricius (1793) in his original description (Fig. 8) as the lectotype of *Papilio sosybius* Fabricius, 1793. It is possible that the Jones illustration may be a composite, amalgamated image of several specimens. If that was the case, the lectotype is the specimen that contributed the most to the illustration. I.e., of all specimens used as models for this possibly composite illustration, the largest number of characters depicted are from the lectotype. As discussed above, our search for this specimen was unsuccessful, and the lectotype is most likely lost. Because we were not able to find definitive wing pattern characters to differentiate between the two eastern US *Hermeuptychia* species (one of which is *Hermeuptychia sosybius* and the other one is not), and the Fabricius description (1793, Fig. 8) augmented with Jones illustration of the lectotype (Fig. 3) is generally consistent with both species, we proceeded with the neotype designation.

### Neotype designation for *Papilio sosybius* Fabricius, 1793

We believe that there is an exceptional need for the neotype to clarify the taxonomic identity of *Hermeuptychia sosybius* and to define which one of the two USA *Hermeuptychia* species this name refers to. We hypothesize that it is more likely that the species from morphogroup 4 – i.e. *Hermeuptychia sosybius* as defined by Seraphim et al. (2014: Table 1 to list its male genitalia characters), characters detailed below – that is widely distributed across eastern US and is more common in collections, is the species that Fabricius named “Papilio Sosybius”. For instance, inspection of *Hermeuptychia* holdings in the USNM collection from 13 US states across its distribution range (MD, VA, SC, GA, TN, AR, AL, KY, MS, LA, TX & FL) revealed that one species outnumbered the other one more than 20 to 1 (169 vs. 8 specimens). The characters seen in specimens of this entity that is significantly more prevalent in collections are consistent with the original description of *Hermeuptychia sosybius* and Jones illustration of the lectotype. Most importantly, the Jones ventral drawing (Fig. 3) shows: 1) a rather straight postmedial brown line on the forewing towards the costa; 2) postmedial brown line on hindwing bulges basad near the costa and 3) it bulges distad somewhat anterior or at the level of the vein M_3_ (should be between large and small eyespots in typical specimens of the more common species, and between two middle small eyespots, closer to the posterior small eyespot, in the rarely collected species). These three characters (indicated in Fig. 68, first image from the left below the line, voucher NVG-1542) are typical of morphogroup 4 specimens. However, the third character (the bulge anterior or posterior of vein M_3_) is somewhat inconclusive from the Jones drawing (Fig. 3) and could possibly be interpreted either way, creating uncertainty with the lectotype identification from the Jones illustration.

In most specimens of eastern US species from a different morphogroup (5, 6, or 7), the forewing postmedial brown line bends basad from vein M_1_ towards the costa, the hindwing postmedial brown line is more straight near the costa, and it bulges distad around vein M_3_ (between the two small eyespots in the middle, closer to the posterior eyespot). While the sample of 21 specimens is too small to evaluate the reliability of the wing pattern characters and even this sample already shows variation in these characters (e.g. in some specimens the forewing line is straight towards the costa), morphogroup 4 species seems to be more consistent with Jones’s lectotype drawing in patterns. Combining this albeit rather weak wing pattern evidence with the 20 to 1 ratio of morphogroup 4 specimens found in collections, its possibly wider distribution across eastern US, and the usage of the name “sosybius” in publications to denote this phenotype and DNA barcode (e.g. Seraphim et al. 2014), we conclude that morphogroup 4 species better represents *Hermeuptychia sosybius* of Fabricius, and look for a neotype specimen of this species.

While this species cannot be confidently identified by wing patterns at the moment, it can be differentiated from other *Hermeuptychia* species by the following combination of male genitalia characters (Figs 60a, d, g, j, 61c, 62o–z2): (1) comparatively large, more gracile and weaker sclerotized (paler) genital capsule (Fig. 60a); (2) medially wider uncus with more prominently convex sides in dorsal (or ventral) view, uncus appears truncated at the apex in dorsal (or ventral) view, but the width of uncus at the apex is generally less than 2/3 of the width of uncus at the narrowest point near the base (Figs 60a, d, 61c); (3) uncus dorsally flatter towards the apex, but convex in lateral view towards the base and with a prominent, thin, membranous carina in basal half (Fig. 60j); (4) valvae elongated, with a saccular lobe, cucullus more gracile, narrower and longer, it projects for close to half of its length farther than the distal end of gnathos (lateral view, Fig. 60g, j); (5) cucullus narrow at the apex, usually with three to five (mostly four) prominent apical teeth (Fig. 60g, j); (6) interior surface of cucullus ventrally without a prominent bulge, best seen in ventral view (Fig. 60d); (7) aedeagus is more gracile, narrower and longer, especially near the distal end, evenly curved or bent distad the middle (Fig. 60d, g, j); (8) longer than wide phallobase (Fig. 60g, j); (9) larger and wider saccus, but shorter than 2/3 of valva length (Fig. 60d). Further analyses and comparisons of genitalia characters between *Hermeuptychia* species are given in Table 1 of Seraphim et al. (2014). In addition, specimens from morphogroup 4 of Seraphim et al. (2014) clustered as molecular group G in the DNA barcode tree. All 40 DNA barcodes we obtained for specimens of the species that we are selecting to represent *Hermeuptychia sosybius*, closely clustered together with the sequences of group G in our trees as well (Fig. 66b).

From the Jones drawing, it is not possible to unambiguously determine the sex of the illustrated specimens because *Hermeuptychia* are not prominently dimorphic sexually, although the darker color of the specimen shown in dorsal view and wing shape on both illustrations is more consistent with a male. We decided to choose a male specimen as the neotype because male genitalia have been used more widely in *Hermeuptychia* taxonomy, were illustrated for the majority of known species by Forster (1964) and extensively analyzed by Seraphim et al. (2014).

The locality of *Hermeuptychia sosybius* types was not stated in the original description and currently remains unknown. However, we could attempt to deduce it by comparative analysis of wing patterns on Jones’s drawings. Large eyespots on both wings, some mostly black and pupilled with pale blue are distinctive. Because the size of eyespots is highly variable in *Hermeuptychia*, it is conceivable that the Drury’s specimens originated in Central or even South America. However, due to very strong development of eyespots and characteristic shape of rusty-brown lines ventrally on both wings, Jones’s drawings are more likely to depict eastern USA *Hermeuptychia*. Most importantly, the name “sosybius” has been applied to these USA populations historically, and in the interest of stability it is best to secure this name for these populations. If the *Hermeuptychia sosybius* types were collected in the USA, it is most likely that Drury obtained them from John Abbot and they originated in the eastern coastal US, possibly in Georgia or Virginia (John V. Calhoun, pers. comm.). Populations of the morphogroup 4 species are continuous and widely distributed in east US (Opler et al. 2013), and they show essentially identical DNA barcode sequences from North Carolina to south Texas (Fig. 66b). Genitalia of inspected specimen do not reveal notable differences across the range either. Recently, Robbins and Lamas (2006) designated a neotype of *Calycopis cecrops* (Fabricius, 1793), a species described by Fabricius in the same publication with *Hermeuptychia sosybius* and under similar circumstances (i.e., Jones illustrations) from “Indiis”, later proposed to be “one of the states along the eastern coast of the United States between Virginia and Georgia, and probably the latter” by Field (1967). Robbins and Lamas (2006) have chosen the neotype to be from USA: Georgia: Chatham Co., Savannah. We could not have done better, and simply follow their example.

**Figures 10–21.**
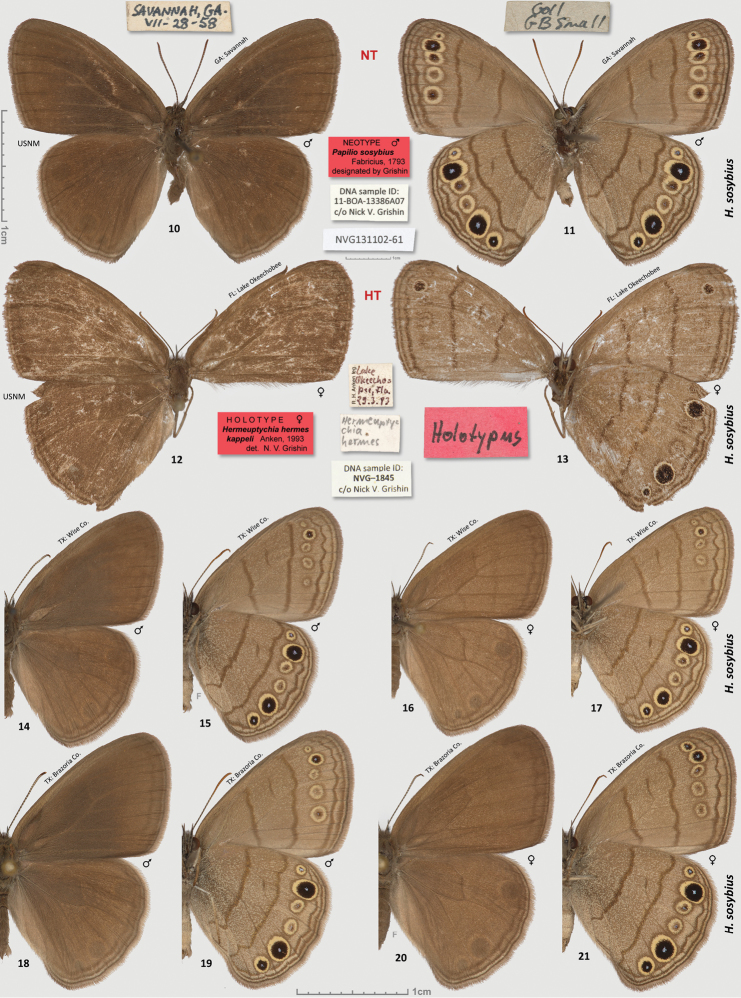
*Hermeuptychia sosybius*. **10**–**11** neotype designated herein and **12**–**13** holotype of *Hermeuptychia hermes kappeli*, data in text **14**–**15** ♂ USA: Texas, Wise Co., LBJ National Grassland, ex ovum, eclosed 3-Aug-1998, leg. N. V. Grishin **16**–**17** ♀ ibid, 10-Aug-1998 **18**–**19** ♂ USA: Texas, Brazoria Co., Bar-X Ranch, Rd. 971N, ex ovum, eclosed 18-Apr-2000, leg. N. V. Grishin **20**–**21** ♀ ibid, 21-Apr-2000. Dorsal/ventral surfaces are in even/odd-numbered figures. Labels are shown for primary types in-line with the specimens and are reduced 2.5-fold compared to specimens as indicated by a smaller scale bar. “F” specifies mirror image (left-right inverted).

A male specimen (Figs 9–11, genitalia Fig. 62p) bearing three rectangular labels: yellowing white, handprinted on one side - || SAVANNAH, GA. | VII-28-58 ||, grayish, handwritten on the other side - || Coll | G B Small ||; white printed - || DNA sample ID: | 11-BOA-13386A07 | c/o Nick V. Grishin ||; white printed - || NVG131102-61 ||; and a plastic glycerin-filled vial with genitalia on the same pin with the specimen, is hereby designated as the neotype of *Papilio sosybius* Fabricius, 1793. Upon this publication, red printed label || NEOTYPE ♂ | *Papilio sosybius* | Fabricius, 1793 | designated by Grishin || will be added. Forewing length of the neotype is 15.5 mm, and this specimen can be recognized by a unique pattern of minor damage to scale cover on wings above, i.e. a longitudinal scratch in the distal half of the left forewing discal cell and a scratch across the discal area of both right wings (Fig. 10). Prior to genitalia dissection, abdomen of the neotype was used to extract total genomic DNA as described in Materials and methods section. The neotype wing pattern mostly agrees with the original description and is similar to Jones illustrations, and the choice of the species is consistent with the usage of this name. The original type locality is not specified in the description (Fig. 8), and the new type locality of *Hermeuptychia sosybius* according to ICZN Article 76.3 (ICZN 1999) is USA: Georgia: Chatham Co., Savannah. The neotype is in the National Museum of Natural History, Smithsonian Institution, Washington, DC (USNM). It is our pleasure to select this excellent specimen collected by Gordon B. Small, one of the most knowledgeable and finest collectors of American, and in particular Panamanian, butterflies (Nicolay 1989), who “knew more about butterflies than any person” (DeVries 1989) and whose exquisite and comprehensive collection of over 50,000 masterfully prepared specimens, rich in rare and undescribed species, is in USNM for future generations to study.

Barcode sequence of the neotype: Genbank accession KJ025561, 658 base pairs:

AACTTTATATTTTATTTTTGGTATTTGAGCAGGAATAATTGGAACATCATTAAGTTTAATTATCCGAATAGAATTAGGTAACCCAGGATTTTTAATTGGAGATGACCAAATTTATAATACTATTGTTACAGCTCATGCTTTTATTATAATTTTTTTTATAGTAATACCTATTATAATTGGAGGATTTGGTAATTGACTTATTCCTTTAATATTAGGAGCTCCTGATATAGCTTTTCCGCGTATAAATAATATAAGATTTTGATTATTACCTCCATCTTTAATTTTATTAATTTCTAGCAGTATTGTAGAAAATGGAAGTGGAACAGGATGAACTGTTTACCCCCCTCTTTCATCTAATATTGCTCATAGAGGTTCTTCAGTAGATTTAGCAATTTTTTCTCTTCATTTAGCTGGAATTTCATCAATTTTAGGAGCTATTAATTTTATTACAACAATTATTAATATACGAATTAATAATATATCTTATGATCAAATACCTTTATTTATTTGAGCTGTAGGAATTACTGCTCTTCTTTTACTTCTCTCATTACCTGTTTTAGCAGGAGCTATTACCATACTTCTTACTGATCGAAATTTAAATACATCATTTTTTGATCCTGCAGGAGGAGGAGATCCTATTTTATATCAACATTTATTT

We believe that our designation of the neotype completely satisfies qualifying conditions of the ICZN Article 75.3 (ICZN 1999). I.e., the exceptional need for the neotype arose due to our discovery that more than one *Hermeuptychia* species was present in eastern USA, and neither the original description, nor the only available illustration of *Papilio sosybius* Fabricius, 1793 lectotype was sufficient to determine which species, if any, was *Hermeuptychia sosybius*. The neotype was designated to clarify the taxonomic identity of *Hermeuptychia sosybius*, i.e., to define which one of the two eastern US *Hermeuptychia* species (than cannot be confidently told apart by the wing patterns) was *Hermeuptychia sosybius*, and to clarify the type locality of *Hermeuptychia sosybius*, which was not stated in the original description (Art. 75.3.1). *Hermeuptychia sosybius* was differentiated from other *Hermeuptychia* species by its DNA barcode given above that placed it in a molecular group G of *Hermeuptychia* species per Seraphim et al. (2014), and by its attribution to the morphogroup 4 by Seraphim et al. (2014), who listed its diagnostic male genitalia characters (Seraphim et al. 2014: Table 1); these characters were elaborated upon and illustrated in this study, e.g., Fig. 60a, d, g, j (Art. 75.3.2). The neotype specimen could be recognized by its labels and appearance as described above and was illustrated in Figs 9–11 (Art. 75.3.3). The reasons to believe that the *Hermeuptychia sosybius* lectotype was lost and the steps we took to trace it were detailed above under the heading “Historical investigations into *Papilio sosybius* Fabricius, 1793" (Art. 75.3.4). We presented the evidence that the neotype was consistent with prior knowledge about *Hermeuptychia sosybius* and was in full agreement with the traditional and current usage of this name (Art. 75.3.5). The neotype specimen came from the general geographic area of hypothesized origin of the lectotype (Art. 75.3.6). Finally, we stated that the neotype is in USNM collection (Art. 75.3.7).

The name “*Hermeuptychia hermes kappeli*” suggested by Anken (1993), type locality “Lake Okeechobee (Nord), Florida, U.S.A.” was regarded as a junior subjective synonym of *Hermeuptychia sosybius* by Calhoun (1997), Lamas (2004) and Pelham (2008). The *Hermeuptychia hermes kappeli* holotype (Table 1, Figs 12–13, USA: Florida: N of Lake Okeechobee, 29-Mar-1983, leg. R. H. Anken, to be deposited in USNM) kindly mailed to us by Dr. R. H. Anken lacks the abdomen, rendering genitalic examination impossible. We obtained a barcode sequence from its legs (Genbank accession KJ025562) to compare *Hermeuptychia hermes kappeli* with other *Hermeuptychia*. The sequence was 100% identical with that of *Hermeuptychia sosybius* neotype (Fig. 66b) and was more than 3.4% different from either the new species, or *Hermeuptychia hermes*. Wing patterns (see discussion below) of the *Hermeuptychia hermes kappeli* holotype were also more consistent with *Hermeuptychia sosybius* than with the new species to be described below. Therefore, *Hermeuptychia hermes kappeli* is either a subspecies of *Hermeuptychia sosybius*, or its subjective junior synonym as previously proposed (Calhoun 1997, Lamas 2004, Pelham 2008). Because DNA barcodes may not vary with subspecies, and we did not study sufficient material from near the type localities of both taxa, we cannot comment on the validity of *Hermeuptychia sosybius kappeli* as a subspecies and adopt the latest treatment (Pelham 2008). However, several butterflies in Florida tend to be regarded as distinct subspecies from nominal taxa with type localities in Georgia or South Carolina (Pelham 2008, Warren et al. 2013). Therefore, a more detailed comparative analysis of wing patterns in *Hermeuptychia sosybius* populations might be desirable.

No other names have been proposed for North and Central American *Hermeuptychia*. Now, after the clarification of the morphogroup 4 species identity by the *Hermeuptychia sosybius* neotype designation and conclusion that *Hermeuptychia hermes kappeli* is either a subspecies or synonym of *Hermeuptychia sosybius*, we can proceed with the description of a different morphogroup (5, 6, or 7) species from southeast Texas.

#### Hermeuptychia intricata

Grishin sp. n.

http://zoobank.org/A89BD0A9-9CE9-4DC7-9EFD-42F77A34B2DD

http://species-id.net/wiki/Hermeuptychia_intricata

Figs 22 –35 , 40–43 , 60c, f, i, l , 61a , 62n , 64i–p , 65 part , 66 part , 67 part , 68 part

##### Description.

**Figures 22–31.**
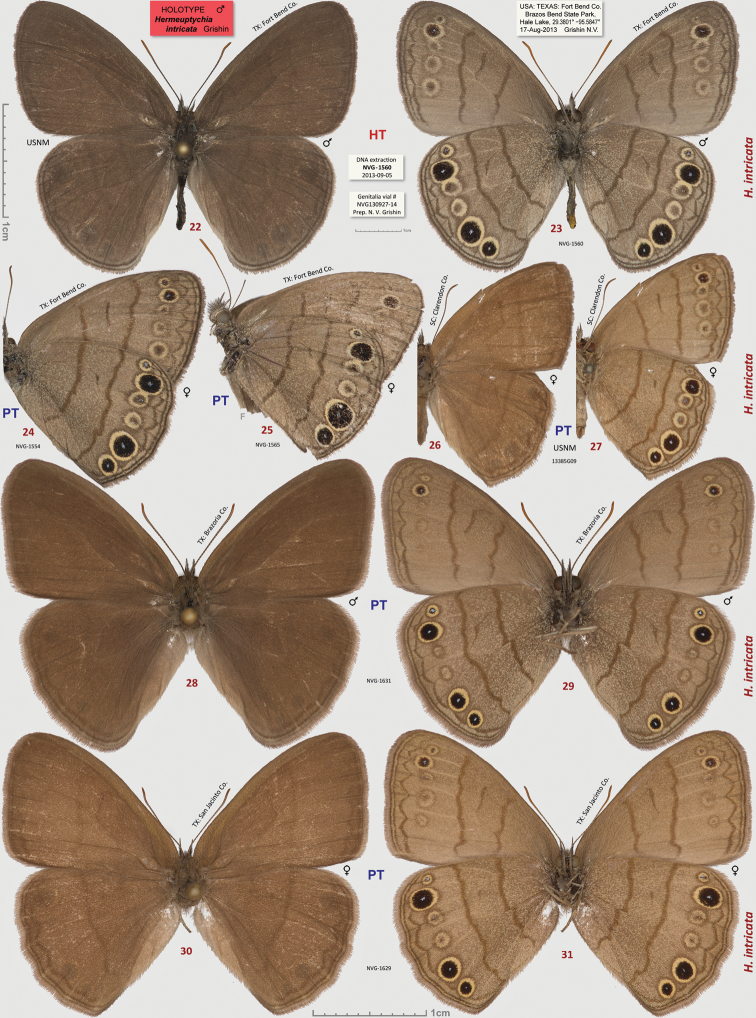
*Hermeuptychia intricata*. **22**–**23** holotype, others are paratypes, data in text and Table 1. Sexes and DNA voucher codes are: **24** ♀ NVG-1554 **25** ♀ NVG-1565 **26**–**27** ♀ 13385G09 **28**–**29** ♂ NVG-1631 **30**–**31** ♀ NVG-1629. Dorsal/ventral surfaces are in even/odd-numbered figures, except **24**, which is ventral. Labels are shown for the holotype and are reduced 2.5-fold compared to specimens as indicated by a smaller scale bar. “F” specifies mirror image (left-right inverted).

**Figures 32–47.**
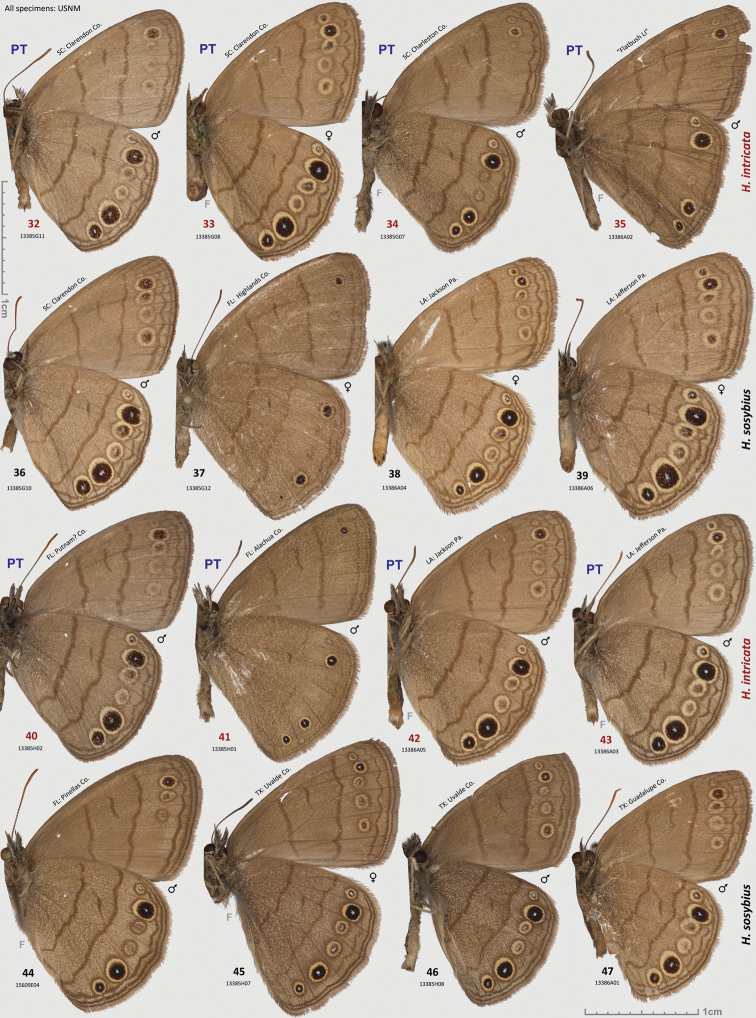
*Hermeuptychia intricata* paratypes and *Hermeuptychia sosybius* specimens. **32–35, 40–43** *Hermeuptychia intricata* **36**–**39, 44–47** *Hermeuptychia sosybius*; data in text and Table 1. Sexes and DNA voucher codes are: **32** ♂ 13385G11 **33** ♀ 13385G08 **34** ♂ 13385G07 **35** ♂ 13386A02 **36** ♂ 13385G10 **37** ♀ 13385G12 **38** ♀ 13386A04 **39** ♀ 13386A06 **40** ♂ 13385H02 **41** ♂ 13385H01 **42** ♂ 13386A05 **43** ♂ 13386A03 **44** ♂ 15609E04 **45** ♀ 13385H07 **46** ♂ 13385H08 **47** ♂ 13386A01. All specimens are in USNM collection. Ventral wing surfaces are shown. “F” specifies mirror image (left-right inverted).

Male (n = 14, Figs 22–23, 28–29, 32, 34–35, 40–43, 68 part) – holotype forewing length = 16.5 mm. Forewing triangular, rounded at apex and tornus, costal and outer margins convex, inner margin almost straight, mildly concave mediad, two discal cell veins bulged at bases, vein 2A thickened basad. Hindwing rounded, almost circular. Wings dorsally dark-brown with sparse olive-beige overscaling and two darker-brown terminal lines. Wings ventrally pale-brown, paler towards inner margin of forewing, with extensive beige overscaling, particularly along veins in distal part in some specimens; submedial and postmedial dark-brown lines and dark-brown end-of-cell streak (smaller on hindwing) between them; forewing postmedial line bent basad near costa in many specimens; hindwing postmedial line almost straight near costa, rarely convex basad and typically convex distad posterior of M_3_ (between the two small eyespots in the middle, closer to posterior eyespot); two terminal dark-brown evenly curved marginal lines, dark-brown sinuous submarginal line, and row of submarginal eyespots basad of the sinuous line and posteriad of outer discal line, largest eyespots black-centered and pupiled with pale-blue scales: on forewing, largest eyespot in cell M_1_-M_2_, eyespot in cell R_5_-M_1_ black-centered in some specimens; on hindwing, largest eyespots in cells Cu_1_-Cu_2_ and M_1_-M_2_, a smaller one in cell Cu_2_-1A+2A, even smaller, but still black-centered and pale-blue pupilled in cell Rs-M_1_, and two smallest, usually without black, but in some specimens pale-blue pupilled eyespots in cells M_2_-M_3_ and M_3_-Cu_1_. Fringes monochrome, a little paler than the ground color of wings. Head, palpi, thorax and abdomen dark-brown above, paler and mostly beige beneath. Antennae dark-brown above with pale scales at segments, orange-brown at the club, beneath beige basad, orange-brown in distal half. Legs brown with beige scales. Male genitalia (n = 14: 12 dissected, 2 inspected *in situ*, Figs 60c, f, i, l, 61a, 62n) – typical for the genus, smaller and darker in color (more sclerotized) than those of *Hermeuptychia sosybius*. Tegumen dome-like, rounded at margins. Uncus leaf-shaped in dorsal view, angled to the sides, roof-like, convex distally but almost flat basally in lateral view, without thin, membranous carina in basal half; apex of uncus pointed, not truncated. Gnathos arms thin, wide apart, divergent, about the same length as uncus. Valvae narrow, elongated with thin cuculli extending past gnathos not farther than a third of their length; cucullus more rounded at apex, usually with a couple of small teeth; cucullus ventrally with inner medial bulge. Saccus about the same length as cucullus, narrow. Aedeagus elongated, almost straight, only slightly and evenly curved, not bent, broader and shorter compared to *Hermeuptychia sosybius*, with a smaller, about as long as wide phallobase. Female (n = 8, Figs 23–27, 30–31, 33, 68 part) – similar to male in facies, with slightly more rounded wings and dorsally paler in color. Female genitalia (n = 8, Fig. 64i–p) with antrum darker in color and smaller than that of *Hermeuptychia sosybius*. Ostium bursae ellipsoidal, its ventral margin longer than dorsal margin. Antrum narrower anteriad, almost triangular in ventral view, somewhat kidney-shaped in lateral view, mostly symmetric. Ductus and corpus bursae each in length similar to antrum; corpus bursae with two signa, spines in a signum broad, leaf-shaped, usually shingled in two rows.

##### Barcode sequence of the holotype.

Genbank accession KJ025595, 658 base pairs:

AACTTTATATTTTATTTTTGGTATTTGAGCAGGAATAATTGGTACATCATTAAGTTTAATTATCCGAATAGAATTAGGTAATCCAGGATTTTTAATTGGAGATGACCAAATTTATAATACTATTGTTACAGCTCATGCTTTTATTATAATTTTTTTTATAGTAATACCCATTATAATTGGAGGATTTGGTAATTGACTTGTCCCTTTAATATTAGGAGCTCCTGATATAGCTTTCCCACGTATAAATAATATAAGATTTTGATTATTACCCCCATCTTTAATTTTATTAATTTCTAGTAGTATTGTAGAAAATGGAAGTGGGACAGGATGAACAGTTTACCCCCCCCTCTCATCTAATATTGCTCATAGAGGTTCTTCAGTAGATTTAACAATTTTTTCACTTCATTTAGCTGGAATTTCTTCAATCTTAGGAGCTATTAATTTTATTACAACAATTATTAACATACGAATCAATAATATATCTTATGATCAAATACCTTTATTTATTTGAGCTGTAGGAATTACAGCTCTTCTTTTACTTCTTTCATTACCTGTTTTAGCAGGAGCTATTACTATACTTCTTACTGATCGAAATTTAAATACATCATTTTTTGATCCTGCAGGAGGAGGAGATCCTATTTTATATCAACATTTATTT

In addition to the holotype, barcodes and ID tags were obtained for 19 paratypes (15 full-length barcodes and 4 ID tags, see Table 1, GenBank accessions: KJ025588–KJ025607, except KJ025595, which is the holotype). Full length barcodes revealed five haplotypes differing from each other by just 1 to 3 base pairs (less than 0.5%). The haplotype of the holotype was more frequently observed (Fig. 66b) and other four haplotypes were confined to a single specimen in the sample.

##### Type material.

**Holotype:** ♂, has the following four rectangular labels: white printed - || USA: TEXAS: Fort Bend Co. | Brazos Bend State Park, | Hale Lake, 29.3801°, -95.5847°| 17-Aug-2013 Grishin N.V. ||; white printed - || DNA extraction | NVG-1560 | 2013-09-05 ||; white printed - || Genitalia vial # | NVG130927-14 | Prep. N. V. Grishin ||; red printed - || HOLOTYPE ♂ | *Hermeuptychia* | *intricata* Grishin ||. The holotype is illustrated in Figs 22–23, 60c, f, i, l, & 68 (first image), and the Genbank accession for its DNA COI barcode sequence is KJ025595. Upon publication, the holotype will be deposited in the National Museum of Natural History, Smithsonian Institution, Washington, DC (USNM). **Paratypes:** 13 ♂♂ and 8 ♀♀, all from USA. Of these, 2 ♂♂ and 5 ♀♀ with the same data as the holotype; and 3 ♂♂ (DNA vouchers: NVG-1541, NVG-1548, & NVG-1551) from 2.5 km to the east, i.e. USA: Texas: Fort Bend Co., Brazos Bend State Park, Horseshoe Lake trail, latitude 29°22'54.96", longitude -95°36'41.06", elevation 15 m, 17-Aug-2013, leg. N. V. Grishin. Sexes and GenBank accessions|DNA voucher numbers|genitalia codes (na if not available) for these paratypes (the same format is used below for others) are: ♂ KJ025588|NVG-1541|NVG131003-03, ♂ KJ025589|NVG-1548|na, ♂ KJ025590|NVG-1551|na, ♀ KJ025591|NVG-1554|NVG130927-07, ♂ KJ025592|NVG-1555|NVG131003-04, ♂ KJ025593|NVG-1556|NVG131003-05, ♀ KJ025594|NVG-1558|NVG130927-08, ♀ KJ025596|NVG-1563|NVG130927-11, ♀ KJ025597|NVG-1565|NVG130927-12, ♀ na|na|NVG131003-10. All but one of these paratypes are illustrated in Figs 24, 25, 68 (above the line). 1 ♂ Texas: Brazoria Co., Bar-X Ranch, Rd. 971N, 29.13252, -95.58340, 7 m, 4-Mar-2000, leg. Nick V. Grishin, KJ025599|NVG-1631|NVG131017-08 (Figs 28–29, 62n). 1 ♀ Texas: San Jacinto Co., Sam Houston National Forest, USF217 @ Big Creek, 58 m, 12-Apr-1998, leg. Nick V. Grishin, KJ025598|NVG-1629|NVG131017-06 (Figs 30–31). 1 ♂ South Carolina: Charleston Co., McClellanville, Wedge Plantation, 6-Apr-1970, leg. D. C. Ferguson, KJ025600|13385G07|NVG131102-38 (Fig. 34). 1 ♀ South Carolina: Clarendon Co., 9-Aug-1898, KJ025604|13385G08|NVG131102-39 (Fig. 33). 1 ♀ ibid., Aug-1910, KJ025605|13385G09|NVG131102-40 (Figs 26–27). 1 ♂ ibid., Aug-1910, KJ025606|13385G11|NVG131102-42 (Fig. 32). 1 ♂ Florida: “Putnam Co | Shell Bluff Landing”, 29-Sep-1985, George Balogh, KJ025602|13385H02|NVG131102-45 (Fig. 40). 1 ♂ Florida: Alachua Co., Gainesville, 12-Mar-1983, leg. Scott W. Gross, KJ025601|13385H01|NVG131102-44 (Fig. 41). 1 ♂ Louisiana: Jefferson Parish, Harahan, 28-Jun-1944, W. D. Field, KJ025603|13386A03|NVG131102-57 (Fig. 43). 1 ♂ Louisiana: Jackson Parish, Jonesboro, na|13386A05|NVG131102-59 (Fig. 42). 1 ♂ “Flatbush LI” (specimen curated in the USNM among *Hermeuptychia* from Louisiana), collected prior to 1941, G. P. Engelhardt Coll., KJ025607|13386A02|NVG131102-56 (Fig. 35).

##### Type locality.

USA: Texas: Fort Bend Co., Brazos Bend State Park, near Hale Lake, latitude 29°22'48.27", longitude −95°35'05.02", elevation 16 m. This locality is by a wooded, partly open, lowland hiking trail (near and along the park paved road) from a parking lot towards the Big Creek, north of the Hale Lake.

##### Etymology.

The name refers to the difficulty in recognizing this very distinct species and its intricate ventral wing patterns. The name is an adjective.

##### Distribution.

Generally, this is a species of eastern US coastal plains and is currently documented from Texas, Louisiana, Florida, and South Carolina (Fig. 67). It is expected to be more widely distributed in the region and the exact boundaries of the range remain to be investigated. For instance, photographs of live individuals from Alabama: Bibb Co., Blue Girth Creek, 08-VIII-2004 & 18-VI-2005 by Vitaly Charny (Warren et al. 2013, specimens not collected, excluded from the type series) exhibit characters more consistent with *Hermeuptychia intricata* than with *Hermeuptychia sosybius* (see discussion below). Furthermore, it is difficult to interpret the locality label for the last listed paratype other than “Flatbush Long Island” [New York, Kings Co.]. However, *Hermeuptychia* has not been recorded that far north–northernmost records are from southern New Jersey and southern Pennsylvania (Opler et al. 2013)–therefore this specimen might have been mislabeled. Nevertheless, searches for this species in the coastal New York/New Jersey area might be interesting to probe its northern distribution limits. An additional specimen (not examined, excluded from the type series) from Costa Rica: Puntarenas Province, GenBank accession AY508548 (Murray and Prowell 2005) has DNA sequence with only 1 bp difference (over 435 base pair C-terminal segment of the barcode) from the USA *Hermeuptychia intricata* barcodes. Unless this sequence is a contamination, it is possible that the Costa Rican specimen is *Hermeuptychia intricata*, which may be ranging southwards at least to Costa Rica. It is apparent, however, that *Hermeuptychia intricata* is either more restricted in distribution and local, or significantly less common than *Hermeuptychia sosybius*, because several dozen available barcode sequences of *Hermeuptychia* specimens from different parts of the range in east US (NC, TN, FL, LA, OK and TX, see Fig. 66b) clearly group with *Hermeuptychia sosybius*, and a sample of 177 genitalically inspected *Hermeuptychia* specimens from 13 US states (MD, VA, SC, GA, TN, AR, AL, KY, MS, LA, TX & FL) in the USNM yielded only 8 *Hermeuptychia intricata* (less than 5%). We hope that a timely description of this species within a few months after its initial discovery will stimulate further studies of this interesting cryptic-in-facies butterfly, which, however, can be easily distinguished from its more common congener by genitalia (Figs 60a, c, d, f, g, i, j, l, 61a, c, 62n–z2 & 64a–p) and DNA barcodes (Fig. 66). All known *Hermeuptychia sosybius* records should be scrutinized in search for *Hermeuptychia intricata*.

##### Diagnosis.

In wing pattern, the new species is very similar to *Hermeuptychia sosybius*. We were not able to find solid diagnostic characters for the new species, and only hypothetical field marks could be suggested (see discussion). However, it could be easily identified by many distinctive characters of genitalia.

Males of the new species possess: (1) smaller and more robust and darker genital capsule, even in males with larger body size (Fig. 60c)–genitalia of *Hermeuptychia sosybius* from various parts of the range are larger and look “wider” and are paler (Fig. 60a); (2) narrower and apically pointed uncus (Fig. 60c, f)–uncus of *Hermeuptychia sosybius* is wider and appears truncated at the apex in dorsal or ventral views (Fig. 60a, d); (3) uncus that is more angled to the sides along the dorsal “rim”, thus appearing “higher” in lateral view (Fig. 60l), but flatter basally due to the lack of prominent carina, vs. a dorsally flatter uncus in distal half, with a well-developed thin, membranous carina in basal half in *Hermeuptychia sosybius* (Fig. 60j); (4) shorter and stouter cucullus, which projects for less than a third of its length farther than the distal ends of gnathos arms (lateral view, Fig. 60i, l)–cucullus in *Hermeuptychia sosybius* is more gracile, narrower and longer, it projects for close to half of its length farther than the distal end of gnathos (lateral view, Fig. 60g, j); (5) cucullus more rounded at the apex, usually with a couple of barely defined, very small apical teeth, vs. three to five (mostly four) larger teeth in *Hermeuptychia sosybius*; (6) interior surface of cucullus ventrally with a more prominent bulge, best seen in ventral view (Fig. 60f vs. 60d); (7) more stout, thicker and shorter penis, best seen in ventral view (Fig. 60f)–penis is more gracile, narrower and longer, especially near the distal end, in *Hermeuptychia sosybius* (Fig. 60d); (8) shorter phallobase, which is about as long as wide (Fig. 60i, l), vs. phallobase that is much longer than wide in *Hermeuptychia sosybius* (Fig. 60g, j); (9) smaller and narrower saccus (Fig. 60f), vs. larger and wider one in *Hermeuptychia sosybius* (Fig. 60d); (10) more obtuse angle formed by the tegumen and vinculum in lateral view (Fig. 60l), vs. typically more acute angle in *Hermeuptychia sosybius* (Fig. 60j).

Females of the new species possess: (I) narrower ostium bursae and smaller, darker antrum (Fig. 64i, j)–ostium bursae and antrum are larger and antrum is paler in color in *Hermeuptychia sosybius* (Fig. 64a, b); (II) ventral margin of ostium bursae that extends farther back than its dorsal margin (Fig. 64k, l)–dorsal margin extends posterior of ventral margin in *Hermeuptychia sosybius* (Fig. 64a, b); (III) antrum that is narrower anteriad, almost triangular in ventral view and symmetric (Fig. 64k, m), vs. rounder, cup-like, slightly asymmetric to the left antrum in *Hermeuptychia sosybius* (Fig. 64e); (IV) more bent antrum, kidney-shaped in lateral view (Fig. 64n), than that of *Hermeuptychia sosybius* (Fig. 64j); (V) signa composed of wider, more flattened and rounder spines, mostly in two rows, vs. narrower spines in three to five irregular rows in *Hermeuptychia sosybius*.

Characters (2) and (3) in males (more pointed apex of uncus and uncus more angled to the sides from the central “rim”) seem to be the easiest to examine without full dissection by brushing the scales off the abdomen tip, even in dry specimens (Fig. 62a, c). Identification of dry females might be more problematic due to abdomen shriveling, however, in freshly caught individuals, ostium bursae and antrum can be easily exposed by squeezing the abdomen in distal third, and the character (II) becomes observable (relative position of ostium bursae margins). Due to these very significant and easily observed differences in genitalia, identification in the field immediately after capture is expected to be straightforward, however, more work remains to be done to discover diagnostic wing pattern characters.

DNA barcodes, consistently with genitalia, set the new species far apart from sympatric *Hermeuptychia sosybius*, and the difference is about 3.5%, which is significantly higher than “a clear threshold for intra- and interspecific mean distances around 2%”, as quoted from the recent comprehensive analysis of *Hermeuptychia* (Seraphim et al. 2014).

While the discovery of this second (and new) *Hermeuptychia* species in eastern USA was very unexpected to us, the next finding is less surprising, although also interesting. Our analysis of DNA barcodes of Texas *Hermeuptychia* revealed that populations from the lower Rio Grande Valley region of Texas (Webb, Zapata, Starr, Hidalgo, and Cameron Counties) form a tight cluster differing by at least 2% from closely clustered barcodes (divergence average 0.09%, standard deviation 0.19%, maximum below 1%) of over 50 *Hermeuptychia sosybius* specimens across its range from North Carolina to Texas (south to Uvalde, Comal, Guadalupe and Brazoria Counties, Figs 66–67). These south Texas (and northeast Mexico) *Hermeuptychia* populations are phenotypically characterized by smaller and more uniformly sized eyespots and more undulated brown lines. This butterfly has been called “*Hermeuptychia hermes*” in some of the recent literature that advocates the presence of two *Hermeuptychia* species in the US (Miller and Brown 1981, brief comment in Neck 1996, Pelham 2008, Warren et al. 2013). However, DNA barcodes clearly and confidently group these populations with *Hermeuptychia sosybius* (Fig. 66a, bootstrap support above 80%, about 2% sequence difference), and *Hermeuptychia hermes* sequences are more than 4% different from either of these [Fig. 66a and Seraphim et al. (2014)]. According to DNA barcodes, *Hermeuptychia hermes* – type locality Brazil: Rio de Janeiro – is in a different species group and clusters with *Hermeuptychia maimoune* (A. Butler, 1870) rather than with *Hermeuptychia sosybius* (Fig. 66a). Analysis of male genitalia agrees with this conclusion. Indeed, genitalia of south Texas specimens are clearly from the morphogroup 4 (i.e. *Hermeuptychia sosybius*) possessing all the characters specified by Seraphim et al. (2014) and are very different from those of *Hermeuptychia hermes* [see Forster (1964) and Seraphim et al. (2014) for illustrations]. Most obviously, *Hermeuptychia hermes* has much longer saccus compared to shorter and more constricted in the middle valvae. Nevertheless, in addition to at least 2% different barcodes, south Texas morphogroup 4 populations differ from eastern *Hermeuptychia sosybius* in facies to the extent that researchers have been treating them as a species distinct from *Hermeuptychia sosybius* (Miller and Brown 1981, Pelham 2008, Warren et al. 2013). Our analysis agrees with this conclusion. Furthermore, we find subtle, but quantifiable, differences in male genitalia between *Hermeuptychia sosybius* and south Texas *Hermeuptychia* populations. Evidence presented above suggests that the name *Hermeuptychia hermes* should not be applied to them. Since currently there are no named species in the *Hermeuptychia sosybius* group [i.e., molecular group G and morphogroup 4 of Seraphim et al. (2014)] other than *Hermeuptychia sosybius*, and south Texas populations fall confidently in the *Hermeuptychia sosybius* group (Fig. 66a), they represent an unnamed species that is described here.

#### Hermeuptychia hermybius

Grishin sp. n.

http://zoobank.org/B719B2F8-D0AD-4995-8372-6AA2FC2116E3

http://species-id.net/wiki/Hermeuptychia_hermybius

Figs 48–59 , 60b, e, h, k , 61b , 62a–m , 63 part , 64q–z , 66 part , 67 part , 70

##### Description.

**Figures 48–59.**
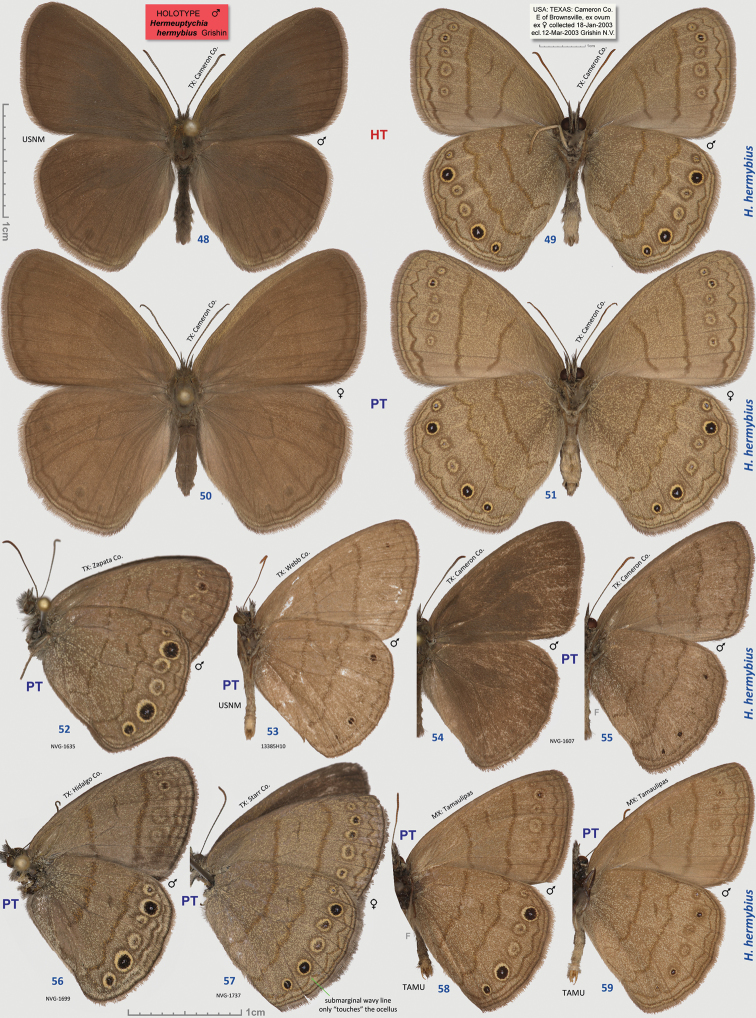
*Hermeuptychia hermybius*. **48–49** holotype, others are paratypes, data in text and Table 1. Sexes and DNA or genitalia voucher codes, or data: **50–51** ♀ USA: Texas: Cameron Co., Brownsville, ex ovum, eclosed 2-Apr-2003, leg. N. V. Grishin **52** ♂ NVG-1635 **53** ♂ 13385H10 **54**–**55** ♂ NVG-1607 **56** ♂ NVG-1699 **57** ♀ NVG-1737 **58** ♂ NVG130104-23 **59** ♂ NVG130104-24. Dorsal wing surfaces are in **48, 50, 54** others are ventral. Labels are shown for the holotype and are reduced 2.5-fold compared to specimens as indicated by a smaller scale bar. “F” specifies mirror image (left-right inverted).

**Figure 60.**
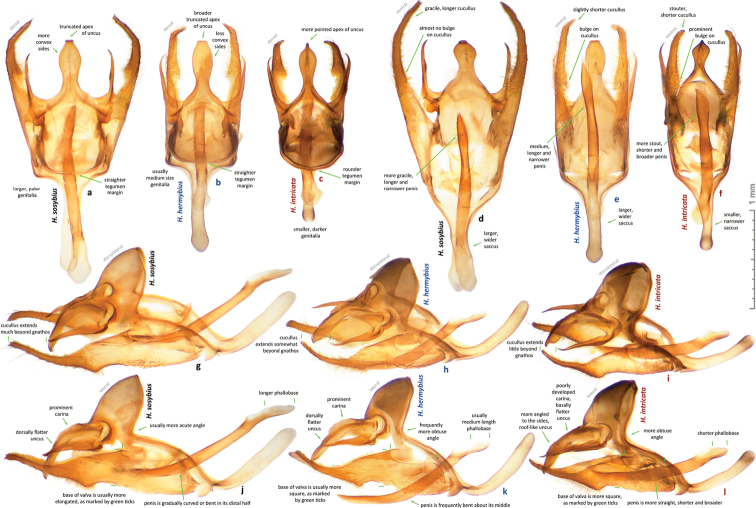
Male genitalia of *Hermeuptychia* from USA: Texas. **a, d, g, j** *Hermeuptychia sosybius*, Fort Bend Co., Brazos Bend State Park, Horseshoe Lake trail, 29°22'54.96", −95°36'41.06", 15 m, 17-Aug-2013, leg. N. V. Grishin, DNA voucher NVG-1542, genitalia NVG130927-03 (forewing length 15 mm) **b, e, h, k** *Hermeuptychia hermybius* sp. n. paratype, Cameron Co., E of Brownsville, 18-Jan-2003, leg. N. V. Grishin, DNA voucher NVG-1607, genitalia NVG130927-18 (specimen Figs 54–55, forewing length 15.5 mm) **c, f, i, l** *Hermeuptychia intricata* sp. n. holotype, Fort Bend Co., Brazos Bend State Park, near Hale Lake, 29°22'48.27", −95°35'05.02", 16 m, 17-Aug-2013, leg. N. V. Grishin, DNA voucher NVG-1560, genitalia NVG130927-14 [USNM] (specimen Figs 22–23, forewing length 16.5 mm). Views: **a**–**b** dorsal, perpendicular to the tegumen-uncus-gnathos plane **c**–**d** ventral, perpendicular to the plane of saccus and valvae (appears larger than dorsal view due to different projection axis) **e**–**f** right dorsolateral **g**–**h** right lateral. All images are to scale. Diagnostic characters are indicated on images. Note that *Hermeuptychia intricata* with larger than *Hermeuptychia sosybius* wings has smaller genitalia.

**Figure 61.**
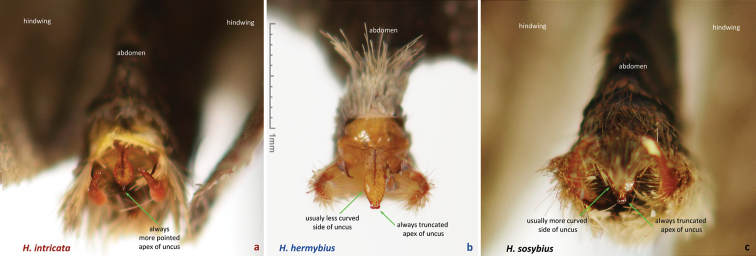
Dorsoposterior view of male abdomens of *Hermeuptychia* from USA: Texas. **a** *Hermeuptychia intricata*, DNA voucher NVG-1548 (mirror image, i.e. left-right inverted) **b** *Hermeuptychia hermybius*, DNA voucher NVG-1635 (also shown in Fig. 62j, specimen Fig. 52) **c** *Hermeuptychia sosybius*, DNA voucher NVG-1553. Data in Table 1. Scales are brushed off the abdomen tip to expose distal parts of genitalia. The easiest to observe character (the shape of the distal end of uncus) is indicated.

**Figure 62.**
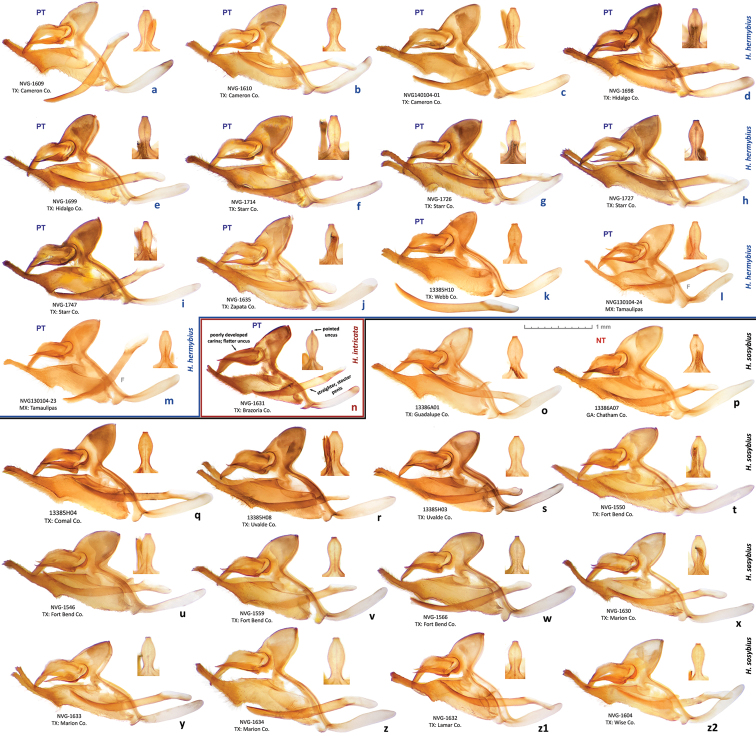
Variation in male genitalia of *Hermeuptychia hermybius* and *Hermeuptychia sosybius*. **a**–**m** *Hermeuptychia hermybius* paratypes, DNA (or genitalia, where DNA sequence is not available, and full data for these given) voucher codes: **a**. NVG-1609 **b** NVG-1610 **c** Texas: Cameron Co., Brownsville {10-13}-Mar-1979, T. Friedlander, NVG140104-01 **d** NVG-1698 **e** NVG-1699 (specimen Fig. 56) **f** NVG-1714 **g** NVG-1726 **h** NVG-1727 **i** NVG-1747 **j** NVG-1635 (also shown in Fig. 61b, specimen Fig. 52) **k** 13385H10 (specimen Fig. 53) **l**–**m** Mexico: Tamaulipas, leg. R. O. & C. A. Kendall: **l** Quintero cave, 7-Jan-1974, NVG130104-24 (specimen Fig. 59) **m** Ciudad Mante, Los Arcos Ct., 19-Dec-1973, NVG130104-23 (specimen Fig. 58) **n** *Hermeuptychia intricata* paratype, NVG-1631 (specimen Figs 28–29), diagnostic characters are indicated on the image **o**–**z2** *Hermeuptychia sosybius*: **o** 13386A01 (specimen Fig. 47) **p** 13386A07, neotype (specimen Figs 9–11) **q** 13385H04 **r** 13385H08 (specimen Fig. 46) **s** 13385H03 **t** NVG-1550 **u** NVG-1546 **v** NVG-1559 **w** NVG-1566 **x** NVG-1630 **y** NVG-1633 **z** Texas: Marion Co., W of Caddo Lake, 5-Apr-1997, leg. N. V. Grishin, NVG-1634 **z1** NVG-1632 **z2** Texas: Wise Co., LBJ National Grassland, ex ovum, eclosed 3-Aug-1998, leg. N. V. Grishin, NVG-1604. **c**, **l**, **m** are in TAMU and **o**–**s** are in USNM collections. Data for most specimens are in Table 1, text, or specified above. Complete genitalia are shown in lateral view, and dorsal view of uncus is shown above and to the right of each specimen. Aedeagus is shown below in **k** DNA (or genitalia, where DNA sequence is not available) voucher codes and general localities are indicated below each genitalia image. “F” specifies mirror image (left-right inverted).

**Figure 63.**
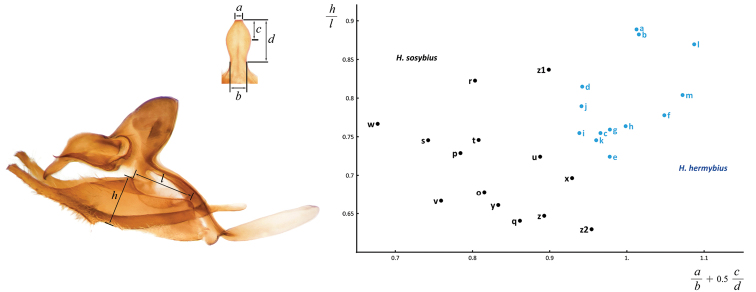
Morphometric differences between male genitalia of *Hermeuptychia sosybius* (black) and *Hermeuptychia hermybius* (blue). Measurements used are marked on dorsal view of uncus (top left) and on lateral view of complete genitalia (bottom left): **a** width of uncus at the apex **b** width of uncus at the narrowest point near the base (“neck” at the joint with tegumen) **c** distance from the uncus apex to the cross-section at the widest point **d** distance from the uncus apex to the cross-section at the narrowest point near the base **l** length of valval dorsal “window” **h** height of valva (in lateral view) at the end of the dorsal “window”, direction of height measurement is perpendicular to the direction of length measurement. Measurements of genitalia shown in Fig. 62 are plotted on the right. Horizontal axis combines all uncus measurements into a formula *a*/*b*+0.5*c/*d* and vertical axis shows measurements of valva as *h*/*l*. Each point corresponds to a specimen and a letter next to it is the same one that denote its genitalia in Fig. 62.

**Figure 64.**
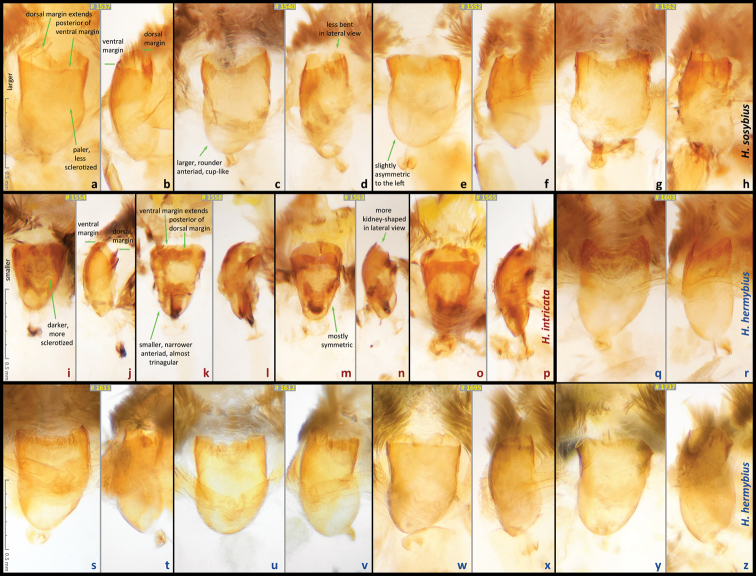
Antrum in female genitalia of *Hermeuptychia* from USA: Texas. **a**–**h** *Hermeuptychia sosybius*, Fort Bend Co., Brazos Bend State Park, 17-Aug-2013, leg. N. V. Grishin: **a**–**f** is from Horseshoe Lake trail, 29°22'54.96", −95°36'41.06", 15 m and **g**–**h** is from near Hale Lake, 29°22'48.27" −95°35'05.02", 16 m; DNA voucher|genitalia dissection codes are: **a**–**b** NVG-1537|NVG130927-01 **c**–**d** NVG-1540|NVG130927-02 (specimen Fig. 12) **e**–**f** NVG-1552|NVG130927-06 **g**–**h** NVG-1562|NVG130927-10 **i**–**p** *Hermeuptychia intricata* sp. n. paratypes, Fort Bend Co., Brazos Bend State Park, near Hale Lake, 29°22'48.27", −95°35'05.02", 16 m, 17-Aug-2013, leg. N. V. Grishin; DNA voucher|genitalia dissection codes: **i**–**j** NVG-1554|NVG130927-07 (specimen Fig. 24) **k**–**l** NVG-1558|NVG130927-08 **m**–**n** NVG-1563|NVG130927-11 **o**–**p** NVG-1565|NVG130927-12 (specimen Fig. 25) **q**–**z** *Hermeuptychia hermybius* sp. n. paratypes **q**–**r** Cameron Co., E of Brownsville, ex ovum ex ♀ captured on 18-Jan-2003, eclosed on 17-Mar-2003, leg. N. V. Grishin, NVG-1603|NVG130927-17 **s**–**t** ibid., eclosed on 14-Mar-2003, NVG-1611|NVG131017-03 **u**–**v** ibid., eclosed on 16-Mar-2003, NVG-1612|NVG131017-04 **w**–**x** TX: Hidalgo Co., 1.5 air mi SE of Relampago, Rio Rico Rd., 26.07, -97.891, 21 m, 19-Oct-2013, leg. W. R. Dempwolf, NVG-1695|NVG131229-03 **y**–**z** Starr Co., 0.5 mi S of Fronton, 26.399, -99.085, 50 m 10-Oct-2013, leg. W. R. Dempwolf, NVG-1737|NVG131229-11 (specimen Fig. 57). Additional data for specimens and their DNA barcodes are in Table 1. In all images, posterior end is pointing up (i.e. ostium bursae is closer to the top of each image); **a, c, e, g, i, k, m, o, q, s, u, w, y** are in lateral view, others are in right ventrolateral view. All images are to scale. Diagnostic characters to tell between *Hermeuptychia sosybius* and *Hermeuptychia intricata* are indicated on images, each character was invariantly observed in all inspected samples of a species, but is indicated (for clarity) on a single image only. We failed to find characters distinguishing female genitalia of *Hermeuptychia hermybius* from *Hermeuptychia sosybius* and simply illustrate genitalic variation.

Male (n = 56, Figs 48–49, 52–56, 58–59) – holotype forewing length = 16 mm. Forewing triangular, rounded at apex and tornus, costal and outer margins convex, inner margin almost straight, mildly concave mediad, two discal cell veins budged at bases, vein 2A thickened basad. Hindwing rounded, almost circular. Wings dorsally dark-brown with sparse olive-beige overscaling and two darker-brown terminal lines. Wings ventrally pale-brown, paler towards inner margin of forewing, with extensive beige overscaling, particularly along veins in distal part in some specimens; submedial and postmedial darker- to rusty- and olive-brown lines and end-of-cell streak (smaller on hindwing) between them; hindwing postmedial line more undulate that in *Hermeuptychia sosybius*, with a stronger bend in M_1_-M_2_ cell; two terminal dark-brown evenly curved marginal lines, dark-brown sinuous submarginal line, more undulate than in *Hermeuptychia sosybius*, barely touching the eyespot in cell Cu_1_-Cu_2_, and row of submarginal eyespots basad of the sinuous line and posteriad of postmedial line, eyespots frequently reduced in size and are more uniformly sized than in *Hermeuptychia sosybius*; usually largest eyespots black-centered and pupiled with pale-blue scales: on forewing, eyespots about the same size, frequently larger posteriad, but eyespot in cell M_1_-M_2_ (usually not the largest in size) and eyespot in cell R_5_-M_1_ (in some specimens) black-centered (more eyespots black centered in some specimens); on hindwing, largest eyespots in cells M_1_-M_2_ and Cu_1_-Cu_2_, a smaller one in cell Cu_2_-1A+2A, even smaller, but still black-centered and pale-blue pupilled in cell Rs-M_1_, and two smallest, usually without black, but in some specimens pale-blue pupilled eyespots in cells M_2_-M_3_ and M_3_-Cu_1_. Fringes monochrome, a little paler than the ground color of wings. Head, palpi, thorax and abdomen dark-brown above, paler and mostly beige beneath. Antennae dark-brown above with pale scales at segments, orange-brown at the club, beneath beige basad, orange-brown in distal half. Legs brown with beige scales. Male genitalia (n = 19, Figs 60b, e, h, k, 61b, 62a–m, 63 part) – typical for the genus, very similar to those of *Hermeuptychia sosybius*. Tegumen dome-like, rounded at margins. Uncus leaf-shaped in dorsal view, almost flat distally but convex basally in lateral view, with a well-developed thin, membranous carina in basal half; apex of uncus appears truncated in dorsal view and sides usually less concave than in *Hermeuptychia sosybius*. Gnathos arms thin, wide apart, divergent, about the same length as uncus. Valvae narrow, but typically broader than in *Hermeuptychia sosybius*, elongated with thin cuculli extending past gnathos usually farther than a quarter of their length; cucullus usually with four apical teeth; cucullus ventrally with inner medial bulge. Saccus about the same length as cucullus, narrow. Aedeagus elongated, bent around its middle, with a medium length phallobase. Female (n = 45, Figs 50–51, 57) – similar to male in facies, with slightly more rounded wings and dorsally paler in color. Female genitalia (n = 9, Fig. 64q–z) as in *Hermeuptychia sosybius*, with pale, yellowish, weakly sclerotized and broad, rounder anteriad, cup-like antrum slightly asymmetric to the left. Ostium bursae ellipsoidal, its ventral margin shorter or equal to dorsal margin. Ductus and corpus bursae each in length similar to antrum; corpus bursae with two signa, spines in a signum narrow, leaf-shaped, placed in three to five irregular rows.

**Figure 65.**
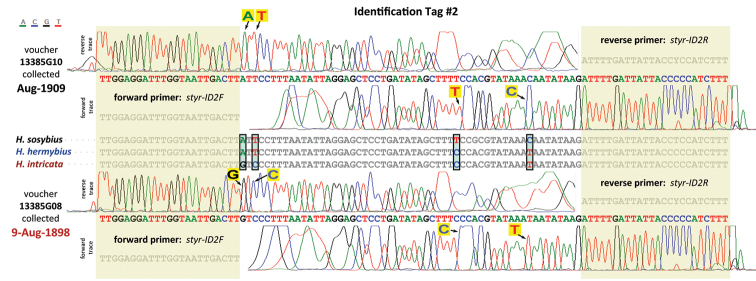
DNA ID tags of specimens that are over 100 years old. ID tag #2 is shown as an example. The tag region sequence alignment of the three species: *Hermeuptychia sosybius*, *Hermeuptychia hermybius*, and *Hermeuptychia intricata* is shown in the middle and positions at which sequences differ are highlighted in cyan and boxed. Each of the three species differs from the other two by at least 2 nucleotides, and *Hermeuptychia sosybius* is different from *Hermeuptychia intricata* by 4 nucleotides. Forward and reverse primer regions are shaded. DNA of the tag was amplified and sequenced in both forward and reverse directions from two over-100-years-old specimens from the same locality (SC: Clarendon Co.). Forward and reverse sequences traces for the first specimen are shown above the reference sequences and the two traces for the second specimen are shown below. It is clear from the traces that the specimen above (13385G10, Fig. 36) is *Hermeuptychia sosybius*, (A, T, T, & C at these 4 positions, no contamination seen) and the one below (13385G08, Fig. 33) is *Hermeuptychia intricata* (G, C, C, & T at these 4 positions and equally unambiguous traces). Nucleotides that identify each specimen are indicated in large letters on yellow background and arrows point to the trace peaks revealing these nucleotides. This strategy was applied to identify 12 very old specimens of three species in a random order and yielded unambiguous identifications for 11 of them. One sample appeared to be contaminated, and the traces showed the presence of several nucleotides in many positions. All 11 DNA-based identifications agreed with genitalic identifications.

##### Barcode sequences.

Full length DNA barcodes were obtained for 19 paratypes (GenBank accessions: KJ025569–KJ025587). The most common haplotype present in 17 sequences (including all 5 barcoded siblings of the holotype) is exemplified by the voucher NVG-1603, Genbank accession KJ025569, 658 base pairs:

AACTTTATATTTTATTTTTGGTATTTGAGCAGGAATAATTGGAACATCATTAAGTTTAATTATTCGAATAGAGTTAGGTAATCCAGGATTTTTAATTGGAGATGACCAAATTTATAACACTATTGTTACAGCCCATGCTTTTATTATAATTTTTTTTATAGTAATACCTATTATAATTGGAGGATTTGGTAATTGACTTATTCCTTTAATATTAGGAGCTCCTGATATAGCTTTCCCACGTATAAATAATATAAGATTTTGATTATTACCCCCATCTTTAATTTTATTAATTTCTAGTAGTATTGTAGAAAATGGAAGTGGAACAGGATGAACTGTTTACCCCCCTCTTTCATCTAATATTGCCCATAGAGGTTCTTCAGTAGATTTAGCAATTTTTTCTCTTCATTTAGCTGGAATTTCATCAATTTTAGGAGCCATTAATTTTATTACAACAATTATTAATATACGAATTAATAATATATCTTATGATCAAATACCTTTATTTATTTGAGCTGTAGGAATTACAGCTCTTCTTTTACTTCTCTCATTACCTGTTTTAGCAGGAGCTATTACCATACTTCTTACTGATCGAAATTTAAATACATCATTTTTTGACCCTGCAGGAGGAGGAGATCCTATTTTATATCAACATTTATTT

**Figure 66.**
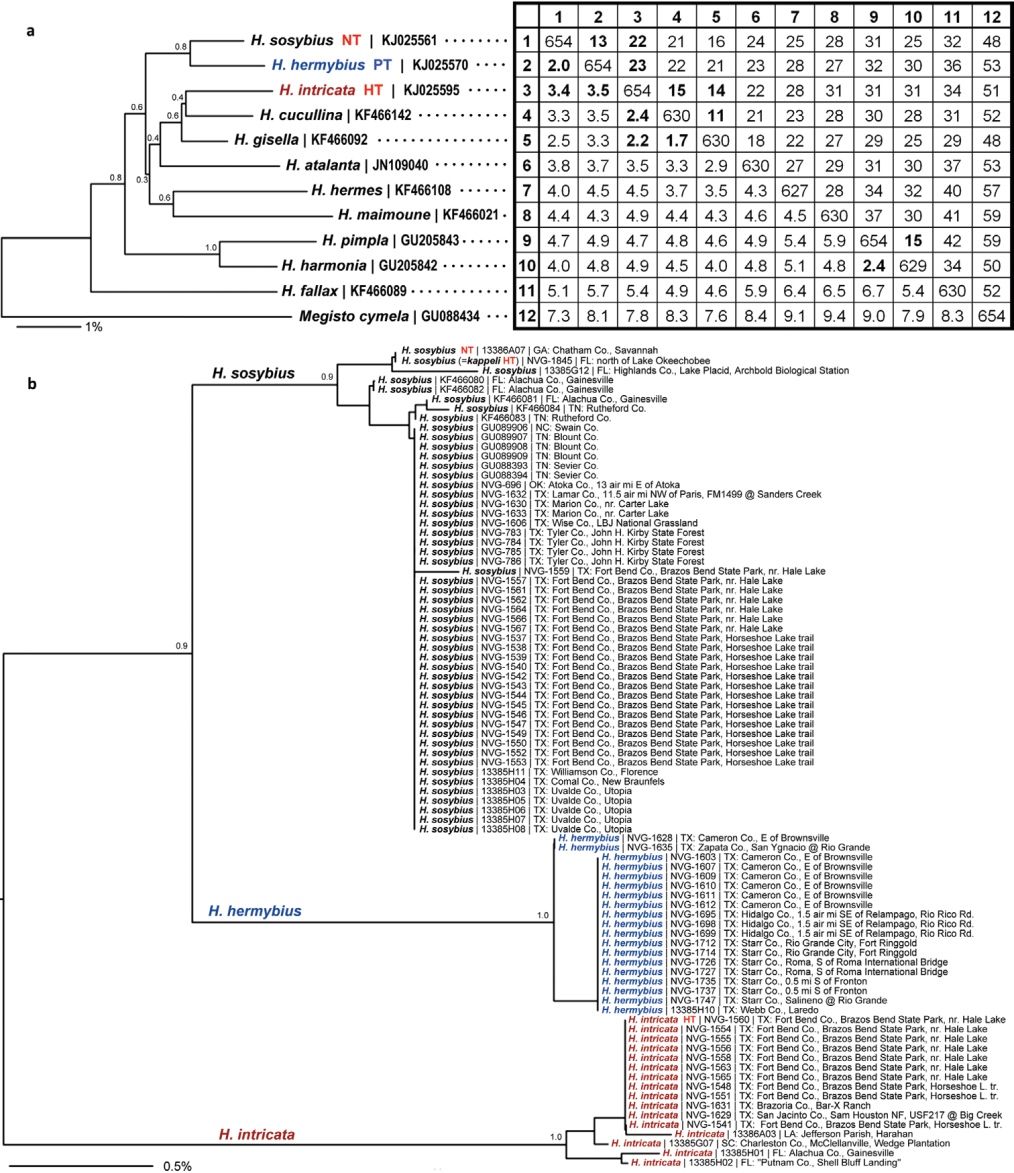
DNA-derived data. **a** Analysis of named *Hermeuptychia* species **b** relationships between *Hermeuptychia* specimens from USA in a form of BioNJ (Dereeper et al. 2008) distance tree. **a** DNA barcode distance matrix is shown on the right and a BioNJ distance tree corresponding to it is on the left. The tree is rooted with *Megisto cymela* (Cramer, 1777) sequence. A more comprehensive tree that includes several specimens of each species (except those described herein) and their detailed analyses are given in Seraphim et al. (2014) and is not repeated here. Only a single representative sequence for each species is used in **a** for clarity. The scale bar corresponding to about 1% difference in sequences is placed below the tree. Bootstrap support values are shown by each node in the tree; values below 0.5 indicate possibly incorrect groupings. GenBank accessions (http://genbank.gov/) for sequences are given after species names. NT, PT and HT refer to neotype, paratype and holotype, respectively. Data for specimens are in Table 1. Specimens 4–11 were not examined and their identification follows that of the authors who performed sequencing studies and analyses (Peña et al. 2010, Silva-Brandão et al. 2011, Seraphim et al. 2014). Percent difference and the number of different nucleotides are shown below and above the diagonal in the matrix, respectively, and the length of each sequence segment (bp) used in the analysis is on the diagonal. Most instructive values discussed in the text are shown in bold font **b** GenBank accession numbers (those that start with letters G or K) for sequences retrieved from GenBank or DNA voucher numbers (those that start from a number or letter N) for sequences obtained in this study and locality data for specimens are given for each sequence. Further details about the specimens are provided in Table 1. *Hermeuptychia sosybius* specimens with sequences obtained from GenBank were not examined and their identification follows that of the authors who performed sequencing studies (Murray and Prowell 2004, Hebert et al. 2010 & Seraphim et al. 2014). All *Hermeuptychia hermybius* and *Hermeuptychia intricata* specimens are paratypes, except the holotype marked with “HT”. Scale bar shown below indicates about 0.5% difference.

The 2 remaining sequences were identical to each other (Fig. 66b) and differed from the sequence shown above by a single base pair (0.15%). Barcode from the oldest and westernmost specimen (TX: Laredo, 15-Apr-1949) was additionally verified with both DNA ID tags as described in Materials and methods section and confirmed to be this species.

##### Type material.

**Holotype:** ♂, has the following two rectangular labels: white printed - || USA: TEXAS: Cameron Co. | E of Brownsville, ex ovum | ex ♀ collected 18-Jan-2003 | ecl. 12-Mar-2003 Grishin N.V. ||; red printed - || HOLOTYPE ♂ | *Hermeuptychia* | *hermybius* Grishin ||. The holotype is illustrated in Figs 48–49. Upon publication, the holotype will be deposited in the National Museum of Natural History, Smithsonian Institution, Washington, DC (USNM). **Paratypes:** 55 ♂♂ and 45 ♀♀, from USA: Texas, unless indicated otherwise. Of these, 9 ♂♂ and 12 ♀♀ are siblings of the holotype read from ova, with the same data, their sexes, eclosion dates and GenBank accessions|DNA voucher numbers|genitalia codes (where available, and in this format for other paratypes) are: 1 ♀ 8-Mar-2003; 1 ♂ 9-Mar-2003, KJ025572|NVG-1610|NVG131017-02 (Fig. 62b); 2 ♂♂ and 1 ♀ 9-Mar-2003; 1 ♂ and 1 ♀ 10-Mar-2003; 1 ♂ and 1 ♀ 11-Mar-2003; 3 ♂♂ 12-Mar-2003; 1 ♀ 14-Mar-2003, KJ025573|NVG-1611|NVG131017-03 (Fig. 64s–t); 1 ♀ 15-Mar-2003; 1 ♀ 16-Mar-2003, KJ025574|NVG-1612|NVG131017-04 (Fig. 64u–v); 1 ♀ 17-Mar-2003, KJ025569|NVG-1603|NVG130927-17 (Fig. 64q–r); 2 ♀♀ 17-Mar-2003; 1 ♀ 21-Mar-2003; 1 ♂ 30-Mar-2003, KJ025571|NVG-1609|NVG131017-01 (Fig. 62a); 1 ♀ 2-Apr-2003 (Figs 50–51). Other paratypes are: 1 ♂ ibid., collected on wing 18-Jan-2003, KJ025570|NVG-1607|NVG130927-18 (Figs 54–55, 60b, e, h, k). 1 ♀ Cameron Co., E of Brownsville, 19-Oct-1997, leg. N. V. Grishin, KJ025575|NVG-1628|NVG131017-05. 1 ♂ Cameron Co., Brownsville, {10-13}-Mar-1979, leg. T. Friedlander, NVG140104-01 [TAMU] (Fig. 62c). 1 ♂ (06-Jun-2007) 1 ♀ (07-Jun-2007) Cameron Co., Los Fresnos, Ted Hunt & Loop Rd., leg. William R. Dempwolf. 4 ♀♀ Hidalgo Co., 1.5 air mi SE of Relampago, Rio Rico Rd., 26.07, -97.891, 21 m, 13-Jun-2013, leg. W. R. Dempwolf; 2 ♂♂ ibid., 19-Oct-2013, KJ025577|NVG-1698|NVG131229-04 (Fig. 62d) and KJ025578|NVG-1699|NVG131229-05 (Figs 56, 62e); 1 ♀ ibid., 19-Oct-2013, KJ025576|NVG-1695|NVG131229-03 (Fig. 64w–x); 3 ♂♂ 4 ♀♀ ibid., 19-Oct-2013; 2 ♂♂ 4 ♀♀ ibid., 21-Oct-2013; 3 ♂♂ ibid., 24-Oct-2013. 1 ♀ TX: Starr Co., Rio Grande City, Fort Ringgold, 26.3707, -98.8064, 45 m, 12-Nov-2010, leg. W. R. Dempwolf; 1 ♀ ibid., 13-Jun-2013; 1 ♂ ibid., 20-Oct-2013, KJ025580|NVG-1714|NVG131229-07 (Fig. 62f); 1 ♀ ibid., 20-Oct-2013, KJ025579|NVG-1712|NVG131229-06; 2 ♂♂ ibid., 20-Oct-2013; 1 ♂ ibid., 23-Oct-2013; 2 ♂♂ 1 ♀ ibid., 9-Nov-2013. 2 ♂♂ Starr Co., Roma, S of Roma International Bridge, 26.4035, -99.0175, 50 m, 20-Oct-2013, leg. W. R. Dempwolf, KJ025581|NVG-1726|NVG131229-08 (Fig. 62g) and KJ025582|NVG-1727|NVG131229-09 (Fig. 62h); 8 ♂♂ 7 ♀♀ ibid., 20-Oct-2013. 1 ♀ Starr Co., Roma Creek, Hwy 650/Hwy 83, 29-Oct-2007, leg. W. R. Dempwolf. 2 ♀♀ Starr Co., 0.5 mi S of Fronton, 26.399, -99.085, 50 m, 20-Oct-2013, leg. W. R. Dempwolf, KJ025583|NVG-1735|NVG131229-10 and KJ025584|NVG-1737|NVG131229-11 (Figs 57, 64y–z); 7 ♂♂ 3 ♀♀ ibid., 20-Oct-2013. 1 ♂ Starr Co., Salineno @ Rio Grande, 26.51463, -99.11633, 53 m, 23-Oct-2013, leg. W. R. Dempwolf, KJ025585|NVG-1747|NVG131229-12 (Fig. 62i). 1 ♂ Zapata Co., San Ygnacio @ Rio Grande, 92 m, 7-Oct-2007, leg. N. V. Grishin, KJ025586|NVG-1635|NVG131017-12 (Figs 52, 61b, 62j). 1 ♂ Webb Co., Laredo, 15-Apr-1949, leg. E. L. Todd KJ025587|13385H10|NVG131102-53 [USNM] (Figs 53, 62k). 1 ♂ Mexico: Tamaulipas: Rt. 101 at Rio Corona, 1-Jan-1980, leg. P. W. Kovarik & D. S. Bogar, NVG140104-04 [TAMU]. 1 ♂ Mexico: Tamaulipas: El Canindo, nr. Ejido San José, 7.5 km W Gómez Farías, 1400 m, {19-21}-Jul-1994, leg. C. Cate & T. Riley, NVG140104-67 [TAMU]. 2 ♂♂ Mexico: Tamaulipas: Ciudad Mante, Los Arcos Ct., 19-Dec-1973, leg. R. O. & C. A. Kendall, NVG140104-22 and NVG130104-23 [TAMU] (Figs 58, 62m); 1 ♂ ibid., 28-Jan-1995, ex larva, foodplant *Panicum maximus* Jacq., NVG140104-24 [TAMU]. 1 ♂ Mexico: Tamaulipas: Quintero cave [22.6333, -99.0333], 7-Jan-1974, leg. R. O. & C. A. Kendall, NVG130104-24 [TAMU] (Figs 59, 62l). 1 ♂ 1 ♀ Mexico: San Luis Potosí: El Salto Falls, 30-Dec-1979, leg. P. W. Kovarik & D. S. Bogar, NVG140104-03 and NVG140104-02 [TAMU].

##### Type locality.

USA: Texas: Cameron County, east of Brownsville. It is a shaded area covered in Guinea grass (*Panicum maximus*), situated near a ravine and overgrown with taller trees.

##### Etymology.

The name is a fusion of two words: herm[es] beginning and [sos]ybius ending. It symbolizes that this species traditionally and previously regarded as *Hermeuptychia hermes* is phylogenetically closer to *Hermeuptychia sosybius*, and yet is distinct from it. The resulting word is unique and currently unknown to internet search engines, which is expected to ease its searches. The name is a noun in apposition.

##### Distribution.

**Figure 67.**
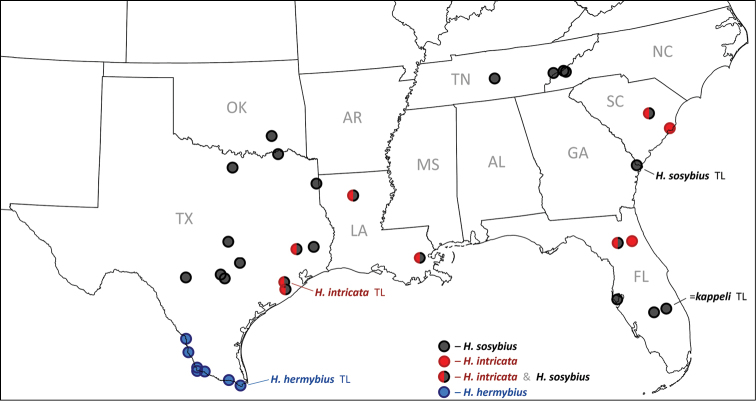
USA localities of *Hermeuptychia* specimens with available DNA barcode information. Color of circles corresponds to species: *Hermeuptychia sosybius* – black; *Hermeuptychia hermybius* – blue, *Hermeuptychia intricata* – red, split red/black circles mark localities where both *Hermeuptychia sosybius* and *Hermeuptychia intricata* were recorded. Type localities are indicated with a corresponding name followed by “TL”. *Hermeuptychia hermes kappeli* was treated as a junior subjective synonym of *Hermeuptychia sosybius* by Pelham (2008). DNA barcode of *Hermeuptychia hermes kappeli* holotype is 100% identical with the barcode of *Hermeuptychia sosybius* neotype. DNA barcode amplification failed for *Hermeuptychia intricata* specimen from LA: Jonesboro and for *Hermeuptychia sosybius* specimen from TX: Brazoria Co., and their identification is based on genitalia only. Specimens from all localities except those from TN and NC (data from GenBank, specimens not inspected) and from FL: St. Petersburg (specimen lacked abdomen) were dissected, and genitalia-based identification agreed with DNA barcode-based identification in all cases (see Fig. 66).

This species is currently recorded from the lower Rio Grande Valley region of Texas along the Rio Grande from Laredo to the Gulf coast (Webb, Zapata, Starr, Hidalgo, and Cameron Counties, Fig. 67) and in neighboring Mexico (Tamaulipas, San Luis Potosí).

**Figure 68.**
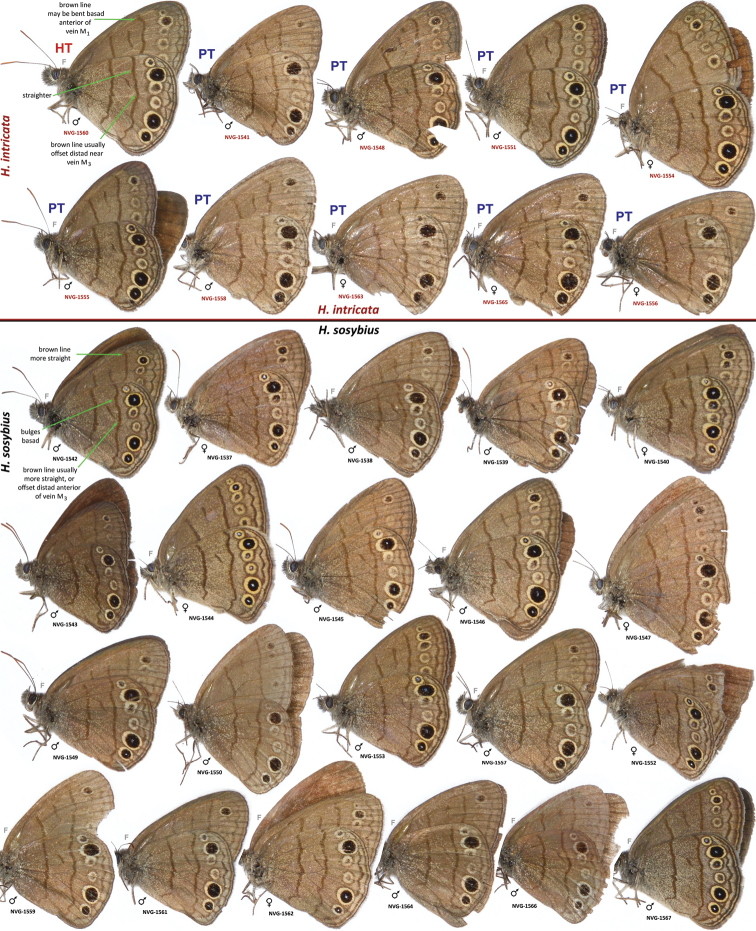
DNA-barcoded *Hermeuptychia* specimens from USA: Texas: Fort Bend Co., Brazos Bend State Park. *Hermeuptychia intricata* is above the line and *Hermeuptychia sosybius* is below the line, photographed prior to removal of body parts for DNA extraction. DNA voucher codes (see Table 1 for data) are shown below each specimen. Hypothetical field marks are indicated on the first specimen of each species. NVG-1537–NVG-1553 are from Horseshoe Lake trail, 29°22'54.96", −95°36'41.06", 15 m; and NVG-1554–NVG-1567 are from near Hale Lake, 29°22'48.27", −95°35'05.02", 16 m, all collected on 17-Aug-2013. Both species are present in each locality. Images are scaled approximately. “F” specifies mirror image (left-right inverted).

##### Diagnosis.

In wing pattern, the new species is most similar to *Hermeuptychia sosybius*, but typically can be differentiated from it by: (a) eyespots that are not only smaller, but also more uniform in size, i.e. out of 5 forewing eyespots, 4 (except the one near costa) are usually about the same size, and the eyespot that is black-ringed in most specimens (second from costa) is typically not the largest (this eyespot is frequently the largest in *Hermeuptychia sosybius*), but the next-to-last eyespot (4^th^ from the costa) is usually the largest one; (b) more undulate postmedial line on ventral hindwing, that frequently strongly bulges basad by the largest eyespot near apex (in cell M_1_-M_2_); (c) more undulate submarginal sinuous line, which on ventral hindwing barely touches the largest eyespot near the tornus (in cell Cu_1_-Cu_2_, second eyespot from tornus, indicated in Fig. 57)–this line is usually fully merged with this eyespot border for some distance in *Hermeuptychia sosybius*. Wing-based identification is not absolute due to extensive pattern variation in both species.

In male genitalia, the new species is also closest to *Hermeuptychia sosybius* and should be attributed to the same morphogroup 4 of Seraphim et al. (2014). It differs from *Hermeuptychia sosybius* in the following trends (Figs 60–61): (1) uncus is less convex and narrower on the sides in dorsal (or ventral) view, with a broader truncated apex, the width at the apex is usually more than 2/3 of the width at the narrowest point near the base (Figs 60b, e, 61b); (2) valva is typically “higher” in lateral view (dorso-ventral direction), more square at the base (Fig. 60k) and is less extended (Fig. 60h); (3) aedeagus is somewhat broader and is frequently bent near its middle, with a medium length phallobase (Fig. 60e); (4) usually more obtuse angle formed by the tegumen and vinculum in lateral view (Fig. 60k). These characters are quite subtle, and as illustrated in Fig. 62 (compare panels a–m with panels o–z2) are subject to significant variation. In contrast, distinction of *Hermeuptychia intricata* (Fig. 62n for comparison) is always definitive and clear-cut. To evaluate the confidence of *Hermeuptychia hermybius* identification by male genitalia and to test the ability to differentiate this new species from *Hermeuptychia sosybius* by objective criteria, we resorted to morphometric analysis (Fig. 63). For simplicity, we have chosen to exploit only two trends listed above: (1) shape of uncus in dorsal view and (2) shape of valva base in lateral view. The shape of uncus was measured by the ratio of width at the apex (*a*) to the width at the narrowest point near the base (*b*), and by the ratio of the distance from apex to the widest point in cross-section (*c*) to the distance from apex to the narrowest point near the base (*d*). We noticed that both of these ratios tend to be smaller in *Hermeuptychia sosybius*. Instead of applying PCA or other similar data-driven technique, which may be biased by the data at hand (i.e. the resulting transformation would change with the dataset used), we combined these measurements in a data-independent transformation. We used a weighted sum of the two ratios, with the weight of the second ratio arbitrarily set to half the weight of the first one: *a*/*b*+0.5*c*/*d*, since the ratio of widths (first ratio) seemed to tell the species apart better than the ratio of lengths (second ratio). The shape of the valva base in lateral view was quantified by the ratio of length of the dorsal “window” (less sclerotized, membranous and flat segment along dorsal side near the base) to the height of the valva at the distal end of the “window”. These variables were measured and computed on a diverse sample of 27 genitalia illustrated in Fig. 62. The resulting plot (Fig. 63 on the right) separated the two species. Therefore these simple measurements could be used to tell between these two cryptic *Hermeuptychia* species by male genitalia. However, we were not able to find characters in female genitalia to differentiate the new species from *Hermeuptychia sosybius*.

Finally, the most confident identification is provided by DNA barcode sequences (Fig. 66) that show little variation within each species (most sequences are identical across the range, maximum difference below 1% in *Hermeuptychia sosybius*), but reveal a definitive 2% hiatus between central and south Texas populations (Figs 66–67). We selected all positions that were invariant in the barcode sample of each species but different between the two species as characters to differentiate *Hermeuptychia hermybius* from *Hermeuptychia sosybius*. The resulting 11 positions are listed in the format “k X (not Y)”, where k is a sequential number of the position (numbering is from 1 to 658 for the barcode sequence shown above as a reference), X is a nucleotide in *Hermeuptychia hermybius* barcodes and Y is a nucleotide in *Hermeuptychia sosybius* barcodes: 64 T (not C), 73 G (not A), 82 T (not C), 118 C (not T), 133 C (not T), 235 C (not T), 238 A (not G), 364 C (not T), 436 C (not T), 526 A (not T), 616 C (not T). These positions distinguish the two species; however, some of the positions are expected to show variation when a larger sample of sequence is accumulated.

##### Life history.

**Figure 69.**
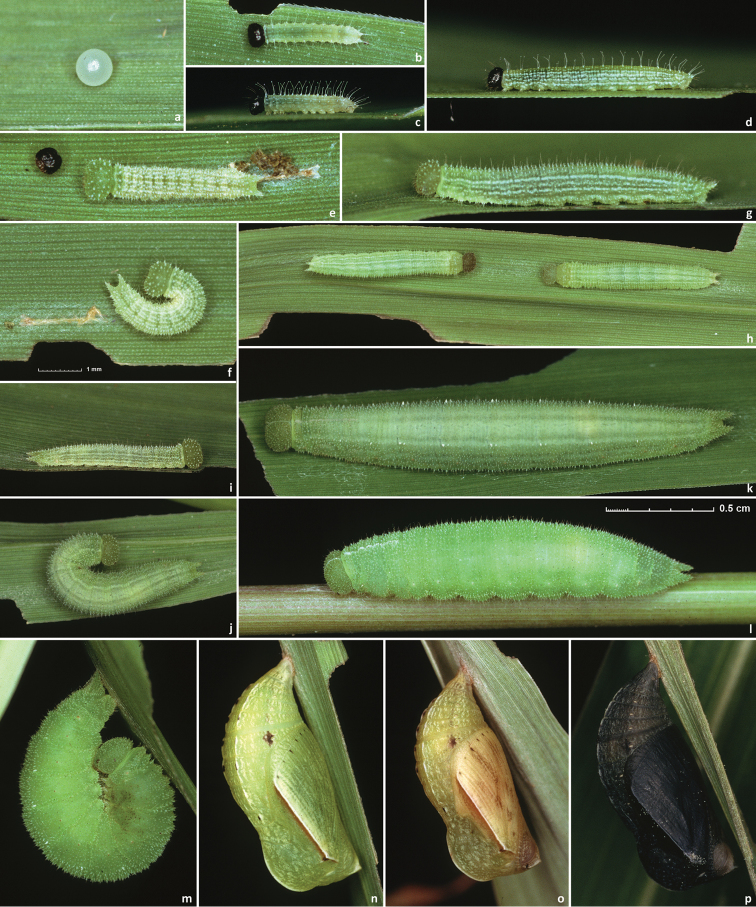
Life history of *Hermeuptychia sosybius*. USA: TX: Brazoria County, Bar-X Ranch, Rd. 971N, 29.13252, -95.58340, ex ovum ex ♀ collected on 4-Mar-2000, except **o**, which is TX: Wise Co., LBJ National Grassland. **a** ovum, 6-Mar-2000 **b**–**d** 1^st^ instars, photographed on 14- 14- & 16-Mar-2000, respectively **e**–**g** 2^nd^ instars photographed on 21- 19- & 21-Mar-2000 **e**, **f** are just after molt, shed larval skins are behind and 1^st^ instar head capsule (black) is on the left in **e**, **f** is in a curled position adopted when disturbed **h** pre-molt quiescent 2^nd^ instar larvae in a typical “head-to-head” resting position, 24-Mar-2000 **i**–**j** 3^rd^ instars, 24- & 27-Mar-2000 **k**–**l** 4^th^ (ultimate) instars, ♂♂, 3- & 6-Apr-2000 **l** close to pupation, note the color and shape change **m** prepupa, 6-Apr-2000 **n**–**p** pupae, 9-Apr-2000, 8-Aug-1998, & 17-Apr-2000 **o** is from Wise Co., wing color is starting to develop **p** near eclosion, dark adult is seen through semi-transparent pupal cuticle. Most images show different individuals. Images **a**–**g** are enlarged 2 times (scale on **f**) compared to the rest (scale on **l**).

**Figure 70.**
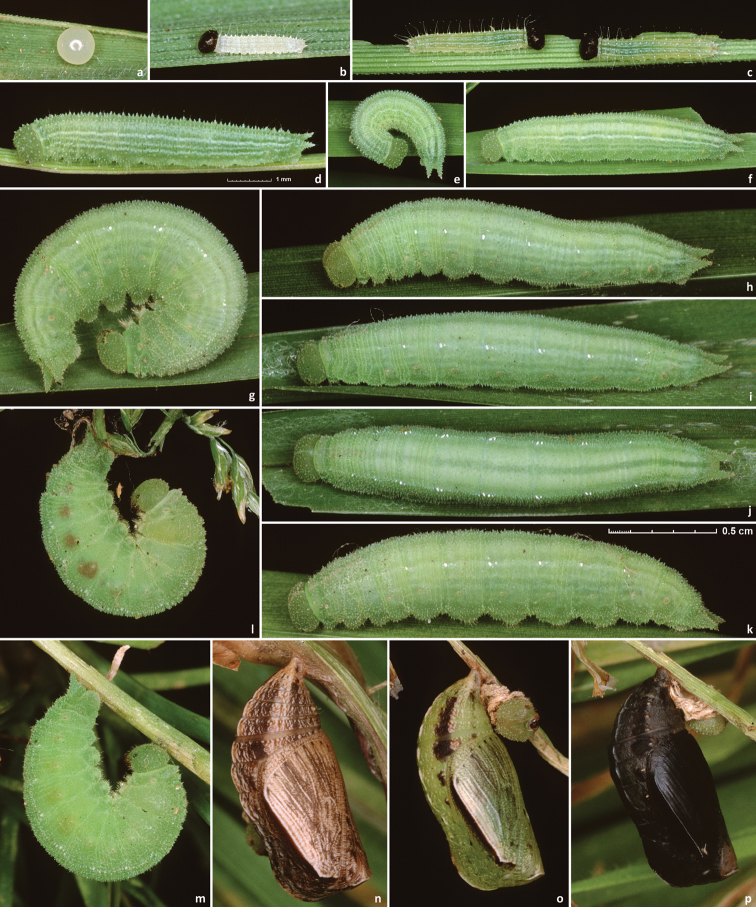
Life history of *Hermeuptychia hermybius*. USA: TX: Cameron County, E of Brownsville, ex ovum ex ♀ collected on 18-Jan-2003. **a** ovum, 23-Jan-2003 **b**–**c** 1^st^ instars, photographed on 30-Jan & 1-Feb-2003, respectively **b** prior to feeding, thus is white in color, **c** shows two caterpillars in a typical “head-to-head” resting position **d** 2^nd^ instar, 10-Feb-2003 **e**–**f** 3^rd^ instars 14- & 15-Feb-2003 **g**–**k** 4^th^ (ultimate) instars, 25- 28- 26- 26- & 25-Feb-2003 **g** is in a curled position adopted when disturbed; **l**–**m** prepupae, 26- & 23-Feb-2003 **n**–**p** pupae, 10-Mar 25-Feb & 4-Mar-2003 **n** is a brown form, shed larval skin is still attached near cremaster in **o** and **p** and is hanging behind the pupa in **n**, **p** near eclosion, dark adult is seen through semi-transparent pupal cuticle. Most images show different individuals, those that eclosed are paratypes. Images **a**–**d** are enlarged 2 times (scale on **d**) compared to the rest (scale on **k**).

The holotype of the new species, along with 21 paratypes are specimen reared in the lab from ova obtained from a captive female. All life history stages are illustrated in Fig. 70, and could be compared to the images of *Hermeuptychia sosybius* life history (Fig. 69). Immature stages of both species are very similar and without larger sample it is difficult to derive solid conclusions about the differences. Nevertheless, the following observations were made. Natural foodplants seems to be *Panicum maximus* (Guinea grass) per R. O. Kendall & C. A. Kendall, who reared caterpillars found on this grass in Mexico: Tamaulipas [TAMU collection]. This plant is also common in the lower Rio Grande Valley and is ubiquitously present where *Hermeuptychia* adults were encountered. Caterpillars hatched from eggs in captivity readily accepted *Cynodon dactylon* (L.) Pers. (Bermuda grass) and were successfully reared on it. Both *Hermeuptychia sosybius* and *Hermeuptychia hermybius* caterpillars go through four instars prior to pupation, and the first instar has black head capsule (Figs 69b–d, 70b–c). In subsequent instars, head capsule is green and round, without horns and projections (Figs 69e–m, 70d–m). Caterpillars of both species typically rest below leaves on loosely made silk pads, frequently in pairs, when two caterpillars face each other “head-to-head” (Figs 69h, 70c). When disturbed, caterpillars first curl into a C head-to-tail while legs being attached to the leaf (Figs 69f, j, 70e), then to a full O, head-to-legs (Fig. 70g). White dorsolateral spots in ultimate instar seem to be more pronounced in *Hermeuptychia hermybius* than in *Hermeuptychia sosybius* (compare Fig. 70g–k with 69k). Pupae of *Hermeuptychia hermybius* were stronger patterned with brown on the sides (Fig. 70o) than those of *Hermeuptychia sosybius* from two distant-from-each-other Texas localities (Fig. 69n–o), and some *Hermeuptychia hermybius* pupae were brown in color (Fig. 70n).

## Discussion

We pose and answer some questions that are likely to arise regarding our description of *Hermeuptychia intricata* and *Hermeuptychia hermybius*.

**Can *Hermeuptychia intricata* be identified by wing patterns alone?** The sample of 20 barcoded and dissected *Hermeuptychia intricata* specimens from the type series (plus 2 more dissected paratypes without barcode sequences) is too small to judge with confidence. At the moment, it seems risky to accept wings-only identification. However, comparative analysis of wing patterns suggests the following three characters that should be investigated further (marked on representative specimens in Fig. 68). First, the postmedial dark-brown line on the ventral hindwing in *Hermeuptychia intricata* bulges distad near the vein M_3_, in between the two smaller eyespots, closer to the posterior eyespot (e.g., compare Figs 32–47, 68). The line is more straight, or sinuous in *Hermeuptychia sosybius*, but frequently with a bulge anterior of the vein M_3_ (closer to large eyespot that is nearer the apex). Second, this line is relatively straight anterior of vein M_2_ in *Hermeuptychia intricata*, but is frequently bulged basad in *Hermeuptychia sosybius*. Third, in some *Hermeuptychia intricata* specimens, the postmedial dark-brown line on ventral forewing bends basad toward the costa (Fig. 68). More precisely, this line bends slightly distad towards the largest eyespot (in cell M_1_-M_2_) and then bends basad from near vein M_1_ (just anteriad of the largest eyespot) to costal margin. In *Hermeuptychia sosybius*, this line is typically straight or even bends distad towards costa. If the line bends basad, the bend is more gradual and begins posterior of M_1_ vein (posteriad of the largest eyespot). However, in some *Hermeuptychia intricata* specimens this bend is not present (Figs 29, 31, 34, 41). If these hypothesized field marks are indeed meaningful, then an individual photographed by Tveten and Tveten (1996: 186) – photograph reproduced with modifications in Brock and Kaufman (2003: 231, rightmost illustration) – is *Hermeuptychia intricata*. Judging from the title of the Tvetens' book, it was photographed in east Texas near Houston, which matches the expected distribution range of this species. Interestingly, another individual shown in Tveten and Tveten (1996: 178) on a full page photograph appears to be typical *Hermeuptychia sosybius*. To facilitate further identification of field marks, we illustrate almost the entire type series of *Hermeuptychia intricata* (Figs 22–35, 40–43, 68), and all DNA barcoded specimens of *Hermeuptychia intricata* and *Hermeuptychia sosybius* from the Brazos Bend State Park, Texas (Fig. 68).

**Are DNA barcode sequences necessary for confident identification of*Hermeuptychia intricata*?** Although the DNA barcoding study has been instrumental in this project, we believe that its conclusions would hold without the knowledge of barcode sequences. Although we first noticed the difference in DNA barcodes, had we dissected the specimens prior to that, the presence of the two species (*Hermeuptychia sosybius* and *Hermeuptychia intricata*) and distinction between them would have become equally clear. We think male and female genitalia offer solid diagnostic characters that are sufficient for confident identification. These characters are numerous, are listed in the diagnosis above, and are illustrated in Figs 60–62 & 64. Therefore, DNA barcode sequences are not required for confident identification of this new species. Nevertheless, barcodes were very valuable to suggest that south Texas *Hermeuptychia sosybius*-like populations with typically smaller, more uniformly-sized eyespots and more undulate ventral hindwing lines are not conspecific with eastern USA populations, but represent a new species, *Hermeuptychia hermybius*. DNA barcodes were equally valuable to confirm that the name *Hermeuptychia hermes* should be best reserved for a South American species (Seraphim et al. 2014).

**Should *Hermeuptychia intricata* be described now from a small sample?** We believe that a description of a new species is an invitation to study it further. The discovery of a new butterfly species in the USA, especially rather distant evolutionarily from other members of the fauna (closest relatives in Bolivia and Brazil), is very exciting and should be made public without delays. It is interesting that the new species is cryptic in wing patterns, and its cryptic nature allowed it to remain unnoticed for over 200 years of research in butterfly taxonomy. Despite the small sample (type series of 22 specimens), we think that our taxonomic conclusions are solid, and genitalic differences are so pronounced that the species status of this taxon is fully justified. However, much remains to be studied, with the most obvious question being the distribution range of *Hermeuptychia intricata*. We hope that our description will stimulate its future studies, including those by citizen scientists and butterfly enthusiasts. Thus we think it is beneficial to describe this new species right away.

**Could *Hermeuptychia intricata* be an extreme variation or a subspecies of *Hermeuptychia sosybius*?** These two taxa are sympatric and synchronic. They could be found flying together at exactly the same spot, with two individuals of different species landing on the same leaf. We think that prominent and easily observable differences in both male and female genitalia are sufficient to strongly support distinction between *Hermeuptychia intricata* and *Hermeuptychia sosybius* as species under essentially any species concept (De Queiroz 2007). Interestingly, even in the absence of barcode sequences, one can associate sexes of these two species correctly by matching morphology of their genitalia: both sexes of *Hermeuptychia sosybius* possess larger (for specimens of equal size) and less sclerotized genitalia, while both sexes of *Hermeuptychia intricata* are characterized by smaller and more sclerotized genitalia. This morphological match of genitalia suggests that the two butterflies are distinct biological species. Additionally, their DNA barcodes differ by 3.5%, which indicates that the lineages were separated from each other by several million years and should be regarded as distinct evolutionary species.

**Could *Hermeuptychia intricata* be a northern subspecies of *Hermeuptychia gisella* or *Hermeuptychia cucullina*?** DNA barcode distance tree (Fig. 66a) shows that *Hermeuptychia intricata* is in the same clade with *Hermeuptychia cucullina* and *Hermeuptychia gisella* (bootstrap support for the clade is about 60%) and is more distant from sympatric *Hermeuptychia sosybius*. Even without the reconstructed tree, analysis of pairwise differences in DNA barcodes (Fig. 66a right, row and column 3 in the matrix) leads to the same conclusion: species closest to *Hermeuptychia intricata* are *Hermeuptychia gisella* (2.2% difference) and *Hermeuptychia cucullina* (2.4% difference). *Hermeuptychia sosybius* appears to be more distant at 3.4% difference. By genitalia, *Hermeuptychia cucullina* is quite different from the other species in its very short, thick and curved penis (Forster 1964, Seraphim et al. 2014). The penis in *Hermeuptychia sosybius* is more gracile. *Hermeuptychia intricata* and *Hermeuptychia gisella* share a shorter, similarly proportioned penis. However, in *Hermeuptychia gisella* the penis is strongly curved (Forster 1964, Seraphim et al. 2014), but in *Hermeuptychia intricata* the penis is bent only slightly, even less than in *Hermeuptychia sosybius*. Interestingly, DNA barcodes suggest that *Hermeuptychia cucullina*, despite its radically different penis, might be closer to *Hermeuptychia gisella* (1.7% difference), and *Hermeuptychia intricata* may be more distant from either of these two species (at least 2.2%, Fig. 66). Although it is not clear whether *Hermeuptychia intricata*, *Hermeuptychia gisella* and *Hermeuptychia cucullina* are sympatric in any locality, differences in their genitalia and DNA barcodes argue that they are three biologically distinct species. However, all three form a species group and possibly are a superspecies (Amadon 1966). A comprehensive comparative study of DNA barcodes and male genitalia morphology by Seraphim et al. (2014) suggested a 2% difference in barcodes of *Hermeuptychia* as a sensible indicator of species distinctness. DNA barcodes of *Hermeuptychia intricata* and *Hermeuptychia gisella* reveal a 2.2% difference. This difference coupled with differences in penis shape allow us to comfortably propose *Hermeuptychia intricata* as a new species.

**Should the neotype for *Hermeuptychia sosybius* be designated now?** It is difficult to derive firm conclusions about taxa without firm identity. We think that it is not prudent to describe a new species sympatric with and hardly separable by wing patterns from *Hermeuptychia sosybius* without clarity about what the name *Hermeuptychia sosybius* stands for. Although the nature of Fabricius types of *Hermeuptychia sosybius* remains unclear and might never come to light, essentially everyone used and uses this name to refer to a common *Hermeuptychia* species widely distributed in the eastern US. Therefore it is sensible to stabilize this meaning by neotype designation. Data we gathered suggests which of the two US species should be *Hermeuptychia sosybius* (in the traditional use of the name) and a specimen of this species was selected as neotype.

**What happened to *Hermeuptychia hermes* for USA populations?** We agree with Seraphim et al. (2014) that the name *Hermeuptychia hermes* should not be applied to USA *Hermeuptychia* populations. Described from Brazil: Rio de Janeiro (either Ilha Rasa or the city of Rio per G. Lamas, pers. comm.), *Hermeuptychia hermes* is characterized by very distinct genitalia, illustrated by Forster (1964: Abb. 60) and is easily distinguished from other *Hermeuptychia* species by valvae strongly constricted in the middle and a very long saccus (see Seraphim et al. 2014 for a photograph). DNA barcodes of specimens with this genitalia type do not group with *Hermeuptychia sosybius*-like barcodes in trees, and are more similar to *Hermeuptychia maimoune* instead (Fig. 66, Seraphim et al. 2014). *Hermeuptychia hermes* barcodes are over 4% different from any of close to 100 available *Hermeuptychia* barcodes from across the US. For instance, *Hermeuptychia hermybius* barcodes from south Texas are much closer to *Hermeuptychia sosybius* barcodes (2% different) than to *Hermeuptychia hermes* barcodes (4.5%). Even *Hermeuptychia intricata* barcodes are closer to *Hermeuptychia sosybius* barcodes (3.4%) than to *Hermeuptychia hermes* barcodes (4.5%, see Fig. 66). The tree topology (Fig. 66a) is consistent with this distance analysis. As a summary, both male genitalia and barcodes indicate that the name *Hermeuptychia hermes* was incorrectly applied to USA populations. Widespread usage of the name *Hermeuptychia hermes* is simply a consequence of it being the oldest and a tendency to lump butterflies similar in wing patterns. Names that are presently considered junior subjective synonyms of *Hermeuptychia hermes* were proposed on the basis of South American specimens (Lamas 2004). While it will be necessary to clarify the status of these taxa by obtaining DNA barcodes or DNA ID tags from the primary type specimens and by designation of neotypes, it is very unlikely that any of these names could refer to USA *Hermeuptychia* populations. As DNA barcodes obtained in Seraphim et al. (2014) show, there is hardly any overlap between species at the northern and southern limits of *Hermeuptychia* range. Therefore, *Hermeuptychia* species from Surname is in all likelihood different from a *Hermeuptychia* species in Mexico.

**Can *Hermeuptychia hermybius* be identified by wing patterns alone?** The *Hermeuptychia hermybius* type series of 101 specimens from all 5 Texas Counties bordering the Rio Grande from Laredo to Brownsville and northeastern Mexico (Tamaulipas & San Luis Potosí) offers excellent material to study variation. This sample suggests that pattern-based identification using the three characters described in the diagnosis above is rather reliable, and *Hermeuptychia hermybius* (in contrast to *Hermeuptychia intricata*) could be mostly identified by wing patterns. Interestingly, while *Hermeuptychia hermybius* is much closer to *Hermeuptychia sosybius* phylogenetically than *Hermeuptychia intricata*, it seems easier to identify by wing patterns. Nevertheless, due to extreme variability of *Hermeuptychia* patterns, some specimens, especially those with smaller, underdeveloped eyespots and other elements of ventral hindwing pattern will not be identifiable by facies.

**Are DNA barcode sequences necessary for confident identification of *Hermeuptychia hermybius*?** *Hermeuptychia hermybius* is a species very close to *Hermeuptychia sosybius*. Our analysis shows that the best characters to tell between the two species are indeed DNA barcodes (Fig. 66). However, we think that wing-based and male genitalia-based (using measurements and graph in Fig. 63) identification will be unambiguous in most cases without knowing the locality of a specimen. Nevertheless, it is likely that some specimens would not be identifiable with confidence in the absence of DNA barcodes.

**Could *Hermeuptychia hermybius* be a southern subspecies of *Hermeuptychia sosybius*?** In our opinion, consistency between the differences in DNA barcodes, wing patterns, male genitalia and historic treatment of south Texas populations as a distinct species by several authors (Miller and Brown 1981, brief comment in Neck 1996, Pelham 2008, Warren et al. 2013), although under the incorrect name “*Hermeuptychia hermes*”, argues for the species status of this taxon. We see a prominent 2% difference hiatus in DNA barcodes between central Texas and south Texas *Hermeuptychia* populations (Figs 66–67) and very little variation in barcodes of *Hermeuptychia sosybius* across its range from North Carolina to Florida and central Texas. This DNA barcode hiatus correlates with the wing pattern differences: smaller and more uniformly sized eyespots, more wavy brown lines in south Texas populations; and with male genitalia differences that were quantified on a diverse sample (Fig. 63). Finally, without knowing DNA barcodes, experienced butterfly taxonomists who were quite familiar with Satyr butterflies, in particular Miller and Brown (1981), listed USA *Hermeuptychia* populations as two species. However, it remains unknown whether *Hermeuptychia hermybius* and *Hermeuptychia sosybius* are sympatric, and the region between the Lower Rio Grande Valley and central Texas should be explored for possible areas of sympatry.

**Is there a specimen age limit for successful DNA barcoding?** The oldest specimens we succeeded with were about 120 years old. These were the oldest specimens we tried. We see that DNA fragments into smaller pieces with age. Therefore, for successful amplification, primers for shorter DNA segments should be designed. Per Materials and methods section, we developed primers for two very short (75 bp and shorter) segments of DNA that contain the highest density of differences between the three US *Hermeuptychia* species. We called these regions ID tags. These tags were successfully and consistently amplified from over 100 years old specimens. Example of the results is shown in Fig. 65. The ID tags allowed us to identify these specimens by DNA and these identifications always matched genitalic identifications. This method could be applied to older specimens and is expected to yield similar success. Potentially, even the oldest preserved butterfly specimen could contain usable DNA that can be extracted and amplified with current methods.

**What could be the English name for *Hermeuptychia intricata* and *Hermeuptychia hermybius*?** Although some object to using “common” names for butterflies (i.e. the names in the native language of a country the insect inhabits), especially in research publications, we believe that common names are beneficial to attract public interest to butterflies and to disseminate knowledge about them more efficiently, thus possibly aiding their conservation. We suggest “Intricate Satyr” as the English name for *Hermeuptychia intricata* to indicate its delicate wing patterns, cryptic nature, difficulty of identification, and the fact that it has remained overlooked in over 200 years of exploration of North American butterflies despite very significant differences in genitalia. We propose the English name “South Texas Satyr” for *Hermeuptychia hermybius* to emphasize its type locality and distribution in the US, and to highlight the importance of South Texas in the studies of butterfly fauna.

## Supplementary Material

XML Treatment for Hermeuptychia intricata

XML Treatment for Hermeuptychia hermybius
